# ‘Iterative Bleaching Extends Multiplexity’ facilitates simultaneous identification of all major retinal cell types

**DOI:** 10.1242/jcs.263407

**Published:** 2024-12-10

**Authors:** Aanandita A. Kothurkar, Gregory S. Patient, Nicole C. L. Noel, Aleksandra M. Krzywańska, Brittany J. Carr, Colin J. Chu, Ryan B. MacDonald

**Affiliations:** ^1^ Institute of Ophthalmology, University College London, London EC1V 9EL, UK; ^2^ Department of Ophthalmology & Visual Sciences, University of Alberta, Edmonton, AB T5H 3V9, Canada

**Keywords:** Immunofluorescence, Zebrafish, Development, Neurons, Glia

## Abstract

To understand the multicellular composition of tissues, and how it is altered during development, ageing and/or disease, we must visualise the complete cellular landscape. Currently, this is hindered by our limited ability to combine multiple cellular markers. To overcome this, we adapted a highly multiplexed immunofluorescence (IF) technique called ‘Iterative Bleaching Extends Multiplexity’ (IBEX) to the zebrafish retina. We optimised fluorescent antibody micro-conjugation to perform sequential rounds of labelling on a single tissue to simultaneously visualise all major retinal cell types with 11 cell-specific antibodies. We further adapted IBEX to be compatible with fluorescent transgenic reporter lines, *in situ* hybridisation chain reaction (HCR), and whole-mount immunofluorescence (WMIF). We applied IBEX at multiple stages to study the spatial and temporal relationships between glia and neurons during retinal development. Finally, we demonstrate the utility of IBEX across species by testing it on the turquoise killifish (*Nothobranchius furzeri*) and African clawed frog (*Xenopus laevis*) to glean large amounts of information from precious tissues. These techniques will revolutionise our ability to visualise multiple cell types in any organism where antibodies are readily available.

## Teaser

**Highlighted Article:** A multiplexed immunofluorescence technique called ‘Iterative Bleaching Extends Multiplexity’ (IBEX) was adapted to label all major cell types in the retina. This technique applied to any multicellular tissue where antibodies are available.

## INTRODUCTION

Tissues are made up of multiple cell types with regional and cell-specific molecular differences. To understand the multicellular composition, and how they are altered during development, ageing and/or disease, we must visualise the complete cellular landscape. This relies on the accessibility of techniques to assay or discriminate between multiple cell types across an entire tissue using their transcriptomes, epigenomes and/or proteomes as features to define cell states or interactions in whole tissues (Choi et al., 2023). However, these techniques remain in refinement and are costly to adapt for individual tissues of choice. As such, developing methods to multiplex existing, optimised and widely available techniques are crucial for maximising cellular studies in any tissue from a wide variety of model organisms. Modern techniques, such as single-cell RNA-sequencing, can identify cell-specific molecular changes in individual cells on a large scale; however, the spatial organisation of these cells is lost in processing and must be mapped back onto the tissue to maximise their value. In contrast, immunofluorescence (IF) techniques allow visualisation of cellular proteins expressed in specific cell types in their undisturbed locations and thus provide spatial information. The number of antibody markers and, by consequence, the amount of information that can be obtained from a single tissue sample is limited by the number of fluorophores that can be imaged at one time. This is usually a maximum of four for most staple laboratory imaging systems and is reduced to three with the addition of a nuclear stain. This is particularly relevant where few antibodies are validated and many are raised in the same host (e.g. rabbit), and so cannot be detected at the same time. Developing techniques to map the expression of multiple proteins onto a tissue will enhance our ability to understand cellular state, behaviour and function in any tissue.

**Fig. 1.**
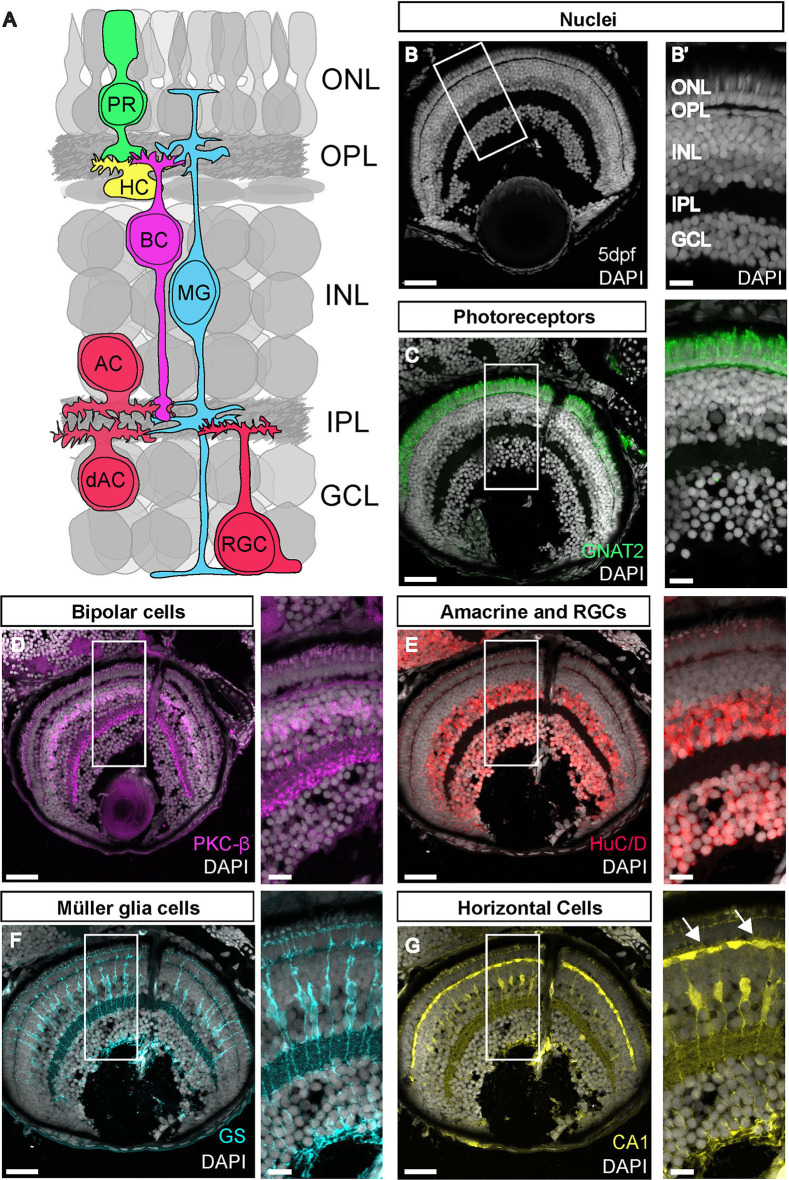
**The retina is made up of highly organised layers composed of neurons and glia.** (A) Schematic of the retina showing the layers and major cell populations with each cell type colour coded. (B) DAPI staining showing the nuclear layers of the retina. (B′) Magnification of B. (C–G) Antibody staining for the main cell types in the zebrafish retina. Arrows in G show horizontal cells. OLM, outer limiting membrane; ONL, outer nuclear layer; OPL, outer plexiform layer; INL, inner nuclear layer; IPL, inner plexiform layer; GCL, ganglion cell layer. All images representative of three experimental repeats. Scale bars: 25 µm (whole retina images); 10 µm (magnified images).

The retina is the light-detecting tissue at the back of the eye, and consists of several different types of neurons and glia. Retinal structure has been well characterised since its early description by anatomists such as Cajal, who used dye labels to identify individual cell types and their organisation based on their unique locations and morphologies (Cajal, 1893). The highly organised retina is made up of five main neuronal cell types and a principal glia cell type called Müller glia (MG) (Fig. 1). Photoreceptors are the light-sensitive cells that synapse onto interneurons (horizontal cells, bipolar cells and amacrine cells), which in turn, relay and modulate the signal and synapse with output neurons (retinal ganglion cells) that connect the retina to visual centres in the brain via the optic nerve. These cells are organised into three discrete cell layers – the outer nuclear layer (ONL), inner nuclear layer (INL) and ganglion cell layer (GCL) – separated by two synaptic neuropils (outer and inner plexiform layers) and form relatively simple circuits (Masland, 2012). The zebrafish retina has been a useful model to study development and disease as it has a conserved organisation and cellular composition with other vertebrates (Avanesov and Malicki, 2010). Furthermore, it is an ideal neural tissue to image *in vivo* as the zebrafish eye is transparent during embryogenesis, develops rapidly [it is functional by 5 days post fertilisation (dpf)] (Patterson et al., 2013) and has a full complement of cell-specific fluorescent reporter lines to visualise every cell type in the tissue (reviewed in Malicki et al., 2016). Furthermore, there are a large number of antibodies with neuronal and glial specificity to discriminate different cell types and visualise morphology in the vertebrate retina (see Yazulla and Studholme, 2001). However, studies of retinal development or disease remain constrained by our limited ability to combine these antibodies to visualise multiple cell types and directly assay cellular relationships or states in the same tissue at one time. Instead, such complex spatial information must be gathered by performing IFs with numerous combinations of the same antibody pools across many tissue sections.

**Fig. 2.**
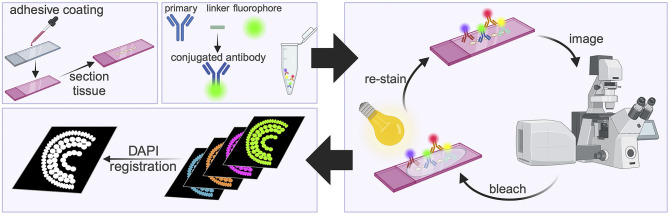
**Schematic of the IBEX method.** Slides are coated with chrome alum gelatin to prevent tissue loss, then tissue is sectioned onto slides. Antibodies are micro-conjugated by mixing the primary antibody with a linker and fluorophore. The antibodies are applied to the slide, incubated, imaged, then bleached using bright light and lithium borohydride before being re-stained. After the imaging rounds are completed, nuclear stains (DAPI) are used to register the image, allowing for all stains to be visualised together. Created in BioRender by Noel, N., 2024. https://BioRender.com/e79p158. This figure was sublicensed under CC-BY 4.0 terms.

Here, we overcome these challenges by adapting ‘Iterative Bleaching Extends Multiplexity’ (IBEX) to the vertebrate retina (Fig. 2). IBEX is a technique developed in mouse and human tissues that allows simultaneous visualisation of up to 60 markers on a single tissue sample (Radtke et al., 2020), thereby providing large-scale, detailed multicellular spatial analysis of tissue. It relies on fluorescently conjugated primary antibodies to enable use of multiple antibodies raised in the same species while avoiding cross-reactivity and permits inactivation of signal to conduct sequential rounds of immunolabelling. First, we validated ‘micro-conjugations’ whereby a small volume of antibody is directly linked to fluorescent dyes to overcome the crucial issue of multiple antibodies raised in the same species. Importantly, these fluorophores can be bleached [photo-chemically inactivated with a combination of bright light and lithium borohydride (LiBH_4_,)] and are compatible with multiple rounds of IF required for the IBEX technique. Using IBEX, we then labelled every major cell type in the retina with 11 specific antibody markers. We enhanced the capabilities of the IBEX technique in zebrafish by pairing with cell-specific transgenic reporter lines, whole-mount IF and *in situ* hybridisation chain reaction (HCR) in zebrafish, by repurposing traditional antigen retrieval methods (sodium citrate incubation at high temperature) to chemically inactivate or ‘quench’ intense fluorophore signal. We also use IBEX to describe the development of two key cell types in the retina – photoreceptors and MG. The techniques described here will be valuable for any tissue and are applicable to any other study where multiplexed IF is required.

Finally, we applied IBEX to two additional aquatic vertebrates. African clawed frog (*Xenopus laevis*) is a well-established model for retinal development and regeneration. However, it is limited by a long generation time and consequent scarcity of adult tissue. Turquoise killifish (*Nothobranchius furzeri*) is an emerging model of rapid ageing with a short fertility period and low fecundity compared to zebrafish. Both models have few transgenic reporter lines available and are currently constrained by the lack of tools to combine several antibodies to make maximum use of precious tissue. Using IBEX to visualise several cell types in a single piece of tissue thus facilitates study of retinal development, ageing and degeneration in various models.

## RESULTS

### Direct conjugation to fluorophores facilitates labelling with multiple antibodies raised in the same host on the same tissue

A major hurdle in IF is labelling with multiple antibodies raised in the same animal (e.g. rabbit) as it would not be possible to distinguish between the antibodies using traditional secondary antibodies. To overcome this limitation, we used ‘micro-conjugation’ reactions (see Materials and Methods) to directly link primary antibodies to distinct fluorophores, avoiding use of secondary antibodies and gaining the flexibility to label each antibody with a fluorophore of choice (Fig. S1). To ensure there was no cross-reactivity or reduction of signal due to competitive antibody binding, we conjugated the rabbit GNAT2 antibody, which labels cone photoreceptors in the zebrafish retina, with four different fluorophores. Using confocal microscopy, we observed robust signal for each of the fluorophores in the photoreceptor layer with no noticeable loss of signal due to multiple conjugated antibodies against the same protein (Fig. S1).

**Fig. 3.**
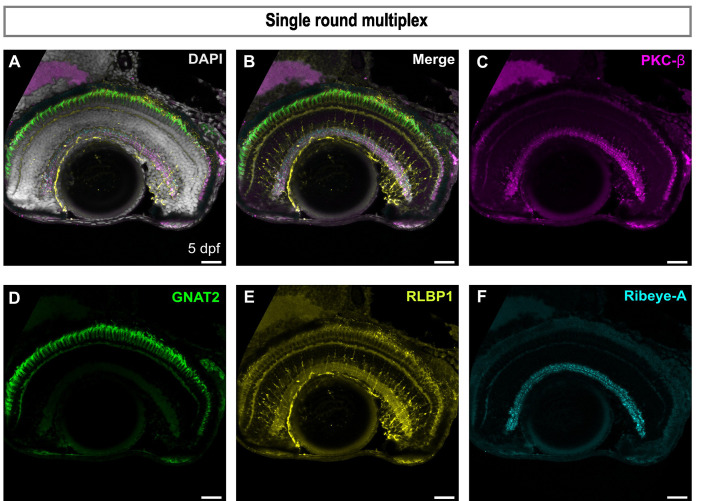
**Direct conjugation to fluorophores facilitates multiple single species antibody labels on the same tissue.** (A) Merged epifluorescence images of a single sagittal retinal section immunolabelled with four antibodies raised in rabbit: PKC-β (magenta), GNAT2 (green), RLBP1 (yellow), Ribeye-A (cyan) and nuclear stain DAPI (grey). (B) Merged image without DAPI. (C) Retinal section immunolabelled with PKC-β, marking bipolar cells. (D) Retinal section immunolabelled with GNAT2 marking cones. (E) Retinal section immunolabelled with RLBP1, marking Müller glia cells. (F) Retinal section immunolabelled with Ribeye-A marking ribbon synapses. All images representative of three experimental repeats. Scale bars: 25 µm.

It is crucial for the multiplexity of the IBEX technique to be able to inactivate the fluorescent signals between rounds of IF. We demonstrate that CoraLite fluorophores can be successfully bleached using lithium borohydride (LiBH_4_) and show a near complete loss of signal and no autofluorescence in any channel post bleaching (Fig. S1). Next, to determine whether we could concurrently label cells with four distinct rabbit polyclonal antibodies, we micro-conjugated each with different fluorophores and conducted a single round of IF and imaging. We labelled cone photoreceptors with GNAT2, bipolar cell ribbon synapses with Ribeye-A, bipolar cell terminals with PKC-β, and MG with RLBP1 (Fig. 3); we were able to visualise these concurrently without any cross reactivity. Therefore, using micro-conjugations we can reliably visualise and inactivate the fluorophores of antibodies raised in the same species on the same tissue section.

### Adapting IBEX to label all major cell types in the zebrafish retina

**Fig. 4.**
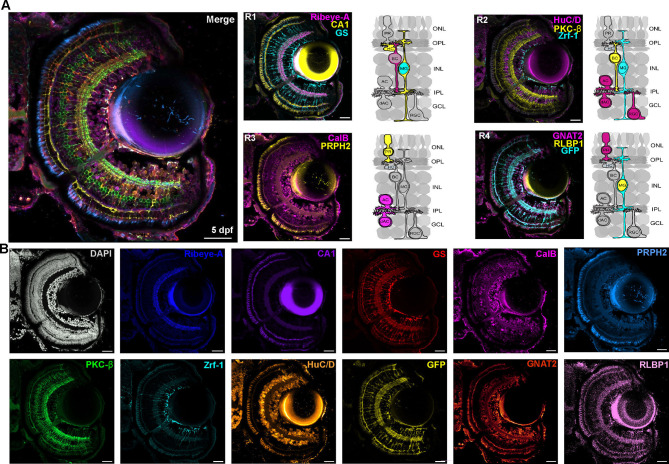
**IBEX enables simultaneous labelling of all major retinal cell types.** (A) Confocal images of 5 dpf Tg(*tp1:eGFP:CAAX*) zebrafish retina showing different rounds of immunolabelling (R1–R4) using IBEX and the merge composite image of each of these rounds. R1 was carried out using Alexa Fluor secondary antibodies, whereas other rounds used microconjugated antibodies. (B) Confocal images showing each antibody used in A to immunolabel a single sagittal retinal section with DAPI and 11 different markers: Ribeye-A (dark blue), carbonic anhydrase (CA1, purple), glutamine synthetase (GS, red), PKC-β (green), Zrf-1 (cyan), HuC/D (orange), GFP transgene (yellow), calbindin (CalB, magenta), peripherin-2 (PRPH2, light blue), GNAT2 (orange) and RLBP1 (pink). All images representative of three experimental repeats. Scale bars: 25 µm.

To label every major cell type in the retina with IF, we designed a panel of markers against proteins expressed in each cell type of the zebrafish retina composed of micro-conjugated antibodies and directly conjugated antibodies. First, we optimised each antibody for use with micro-conjugation by testing for bright, specific labelling in single IF tissue staining and bleaching (see Materials and Methods). In some cases, strongly expressed antibodies were not successfully bleached by LiBH_4_. Fortuitously, an additional antigen retrieval step using sodium citrate was sufficient to quench the staining for these antibodies. Using three rounds of iterative bleaching followed by standard confocal imaging, we labelled each major cell type in the retina using 11 markers and DAPI in the same tissue section (Fig. 4; Movie 1). In each IF round, we used DAPI to label nuclei, which is used for alignment and the ultimate integration of multiple markers on the same tissue, as it does not bleach. This provides a consistent fiducial landmark for image registration. The open source SimpleITK registration software (Radtke et al., 2020) for registration of confocal *z*-stacks is effective at increasing the alignment of DAPI signal between the three rounds (Fig. S2) and allows channels from the different rounds to be merged. As such, we developed panels of combinatorial fluorescent antibody labels against each cell type in the retina, imaged each panel in successive imaging rounds after inactivation of fluorophores and integrated the data onto a single image file (Fig. 4A). To increase the rate at which we could acquire data from multiple samples, we also optimised IBEX and the panel of markers for an epifluorescence imaging system with onboard deconvolution (Leica THUNDER). This allows for a large area of tissue to be imaged quickly and effectively over multiple rounds to visualise nine antibodies and a lectin stain on the same tissue (Fig. S3).

### IBEX is compatible with cell-specific transgenic reporter lines

We aimed to assess whether we could combine IBEX with existing transgenic lines to enhance our multiplex toolbox. To incorporate transgenic reporter lines into the IBEX technique, it is ideal for the fluorescent protein to bleach and make the channel available for future imaging rounds. We tested whether endogenous fluorescent protein signals could be inactivated and then re-labelled with fluorescent protein-specific antibodies by IF. The antigen retrieval step in traditional IF inadvertently inactivates signal of some transgenic lines, which are then boosted with an antibody against the fluorescent protein. We took advantage of this step to test inactivation of transgene fluorescence using several transgenic lines containing cytosolic [Tg(*GFAP:GFP*)*,*Tg(*vsx1:GFP*)^nns5^, Tg(*TP1:Venus-Pest*),Tg(*ptf1a:dsRed*)^ia6^,Tg(*rho:YFP*)^gm500^] or membrane-targeted [Tg(*tp1bglob:eGFP-CAAX*)] fluorescent proteins. We found that both cytosolic and membrane-tagged GFP were quenched after antigen retrieval methods (Fig. S4). These transgenes could then be boosted with an anti-GFP antibody and imaged in the last round of labelling. We confirmed that the transgene retained its cell specificity during this process by co-labelling and observing overlapping labelling with a cell-specific antibody, PKC-β for Tg(*vsx1:GFP*) and glutamine synthetase (GS) for Tg(*tp1bglob:eGFP-CAAX*) (Fig. S4A″,A‴,B″,B‴). However, we could not inactivate the RFP or YFP transgenic lines with LiBH_4_ in combination with intense light nor sodium citrate antigen retrieval (Fig. S4C,D).

### IBEX can be combined with fluorescent *in situ* hybridisation

**Fig. 5.**
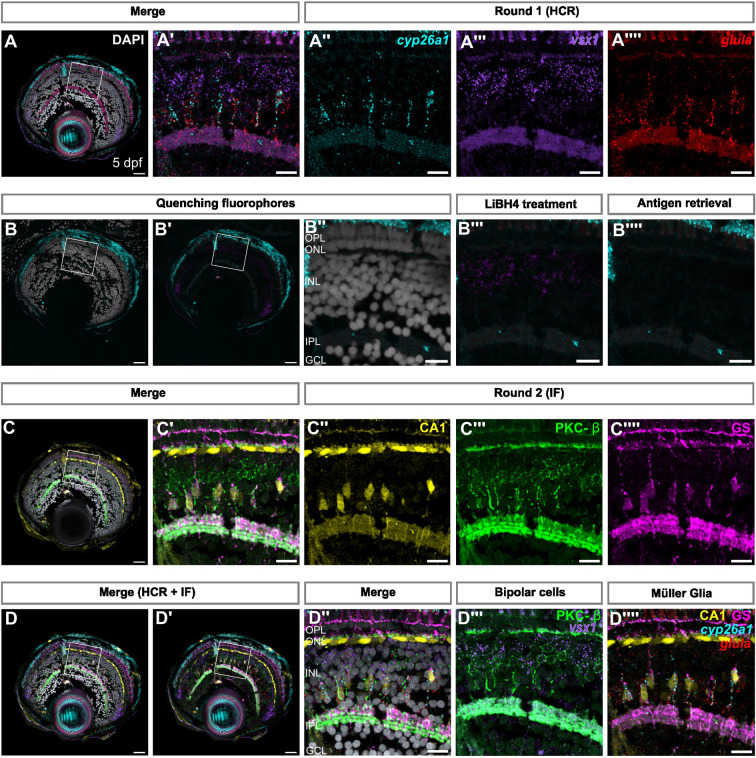
**IBEX is compatible with fluorescent *in situ* hybridisation chain reaction.** (A) Confocal images of retinal sections showing mRNA expression of *cyp26a1*, *glula*, and *vsx1*, using *in situ* HCR. (A′–A⁗) Magnification of the region of interest indicated in A. (B–B‴) Confocal images showing reduced signal of Alexa Fluor 488 and Alexa Fluor 647, but not Alexa Fluor 555 after LiBH_4_ treatment. (B⁗) Heating in sodium citrate at 60°C causes inactivation of Alexa Fluor 555 as well as Alexa Fluor 488 and Alexa Fluor 647. (C) Confocal images of retinal sections immunolabelled with CA1 (yellow), PKC-β (green) and GS (magenta). (C′–C‴) Magnification of the region of interest indicated in C. (D) SimpleITK registered image, showing overlay of both rounds of imaging, and overlay of *in situ* probes and antibodies detecting Müller glia and bipolar cells, respectively. (D″–D‴) Magnification of the region of interest shown in D and D′. GCL, ganglion cell layer; IPL, inner nuclear layer; ONL, outer nuclear layer; OPL, outer plexiform layer. All images representative of three experimental repeats. Scale bars: 25 µm (whole retina); 10 µm (magnified images).

Antibodies specific for a cell type or protein of interest can be limited in zebrafish. As an alternative, *in situ* hybridisation chain reaction (HCR) is a robust method to label mRNA of interest in zebrafish (Choi et al., 2010, 2016, 2018). HCR has been previously combined with IF (Howard et al., 2021; Ibarra-García-Padilla et al., 2021; Ćorić et al., 2023); however, this technique is limited by the number of channels available in a single labelling round on standard microscopes. With a view to overcoming this limitation, we tested whether *in situ* HCR methods to label mRNA would be compatible with IBEX, such that we could conduct an HCR followed by bleaching, IF and integration of labelling techniques on the same retina. For this, we performed HCR for three genes of interest: *cyp26a1*, *glula* and *vsx1.* The expression of these genes is known to be specific to different retinal cell populations: MG (*cyp26a1* and *glula*) and bipolar cells, both ON and OFF (*vsx1*) (Yazulla and Studholme, 2001) (Fig. 5A–A⁗). We then attempted to bleach the signal of these fluorophores using LiBH_4_ treatment. However, we did not observe a significant reduction in signal for Alexa Fluor 555 (Fig. 5B–B‴). We were able to quench this signal using the antigen retrieval technique (Fig. 5B⁗) before conducting a subsequent round of IF with MG antibody markers (CA1 and GS) and the ON bipolar cell marker (PKC-β) (Vitorino et al., 2009) (Fig. 5C–C⁗). We overlaid these two rounds of imaging, one HCR and one IF, which allowed us to visualise expression of the three transcripts of interest and confirm colocalisation with different retinal cell populations labelled by antibodies (Fig. 5D–D⁗). However, it should be noted that the proteinase K used during the HCR protocol led to tissue degradation, as seen by loss of nuclei, which can limit the number of rounds possible with this combination of techniques.

### Whole-mount IBEX facilitates whole-tissue labelling in zebrafish

**Fig. 6.**
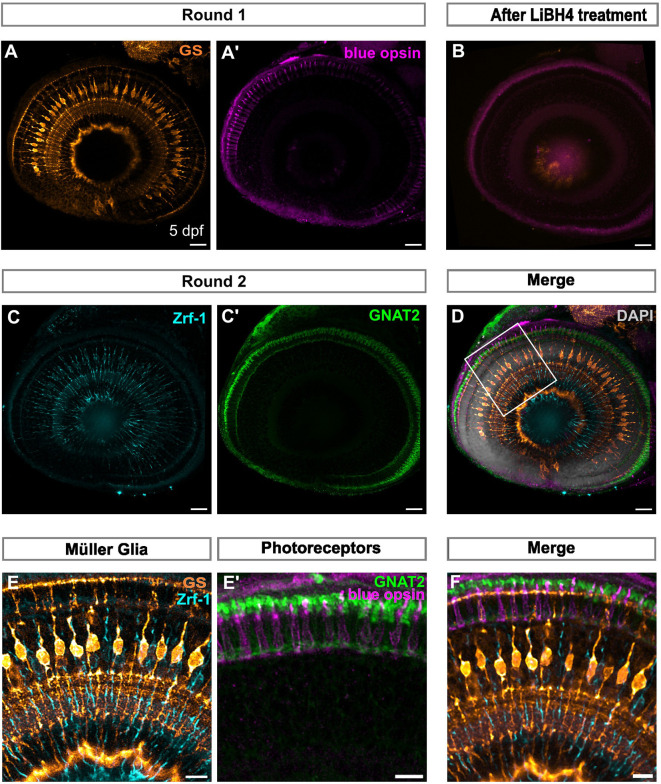
**Whole-mount IBEX facilitates whole tissue labelling in zebrafish.** (A,A′) Confocal images of whole-mount zebrafish larvae at 5 dpf immunolabelled with GS (orange) and blue opsin (magenta). (B) Tissue after bleaching with LiBH_4_ showing reduced signal of the fluorophores CoraLite 488 and CoraLite 647. (C,C′) Confocal images of the second round of immunolabelling to detect Zrf-1 (cyan) and GNAT2 (green). (D) Merge of both rounds of immunolabelling using SimpleTK registration pipeline counterstained with DAPI (grey). (E) Overlap of MG markers GS and Zrf-1 across round 1 and 2. (E′) Overlap of photoreceptor markers blue opsin and GNAT2 across round 1 and 2. (F) Magnification of area highlighted in D, showing overlay of MG and photoreceptor labelling. MG, Müller glia. All images representative of three experimental repeats. Scale bars: 25 µm (whole retina); 10 µm (magnified images).

The relatively small size of the zebrafish retina and the ability to treat the fish to make them optically transparent lends itself to whole-mount immunofluorescence (WMIF) (Inoue and Wittbrodt, 2011; Santos et al., 2018). This technique facilitates the study of cell structure and shape in its native conformation, and overcomes the potential disruption of cell morphology and tissue damage introduced by cryosectioning. However, the hurdle of visualising multiple cell types in the same sample remains. Therefore, we tested whether the micro-conjugated antibody staining is compatible with the thicker tissues in WMIF before carrying out the IBEX protocol. We tested the protocol by immunolabelling MG and photoreceptors with two different antibodies each (GS and Zrf-1 for MG, and GNAT2 and Blue opsin for photoreceptors), over two successive rounds of imaging (Fig. 6A,A′,C,C′). Micro-conjugated antibodies penetrated the tissue and specifically labelled photoreceptors and MG. Treatment with LiBH_4_ successfully bleached the signal of each of the fluorophores between rounds (Fig. 6B). The SimpleTK registration software allowed us to combine the images and observe the colocalisation of antibodies labelling Müller glia and photoreceptors, respectively, across different rounds of IF (Fig. 6E,E′,F; Movie 2). Therefore, WMIF when combined with IBEX allows 3D labelling of multiple cell types and alignment of their spatial relationships to one another between rounds.

### IBEX facilitates the characterisation of retinal histogenesis and patterning

The retina has a stereotyped histogenesis whereby retinal neurons and glia are born and specified in distinct temporal sequence during retinogenesis (Agathocleous and Harris, 2009). Specification of the zebrafish retina begins at 24 h post fertilisation (hpf) as a retinal primordium, completing histogenesis by 73 hpf (Easter and Nicola, 1996) with robust vision beginning at 5 dpf. We used this well-characterised developmental pattern to determine the utility of IBEX to describe cellular morphologies in the highly dynamic developing retina. We focussed on two main cell types – photoreceptors, which have five distinct subtypes that are challenging to visualise simultaneously by traditional methods, and MG, owing to their highly dynamic morphological changes across retinal development. We used cryosections at different key timepoints of retinal development to accomplish this.

**Fig. 7.**
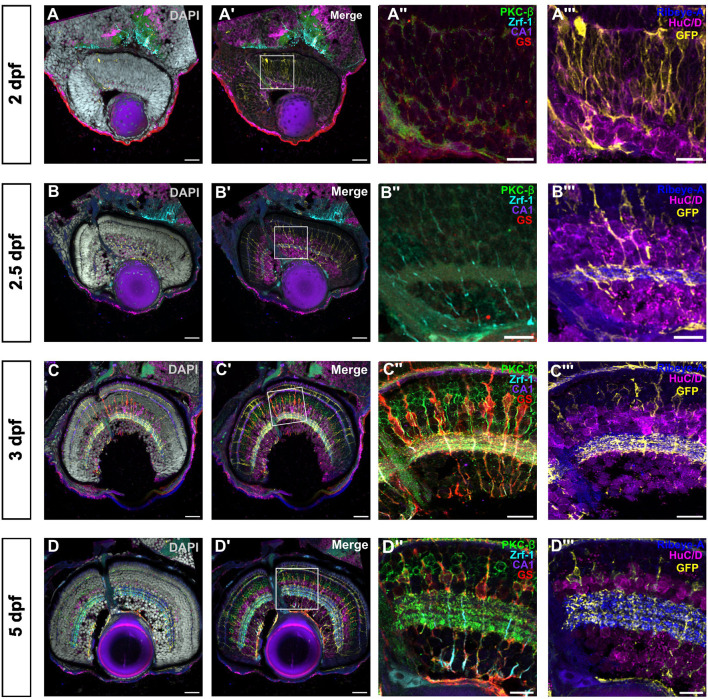
**Visualisation of glial and neuronal development in the zebrafish retina.** Confocal images of the developing zebrafish retina from 2 dpf to 5 dpf immunolabelled with DAPI and seven different markers using IBEX over 3 rounds of immunolabelling with IBEX. A,B,C,D show a merge of all seven markers with nuclear stain DAPI; A′,B′,C′,D′ show the same overlay of markers without DAPI. A″,B″,C″,D″ show the first two rounds of immunolabelling of bipolar cells (PKC-β, green), glial intermediate filaments (Zrf-1, cyan), horizontal cells (CA1, purple) and Müller glia (GS, red) at different timepoints. A‴,B‴,C‴,D‴ show the last round of immunolabelling of ribbon synapses (Ribeye-A, blue), amacrine and ganglion cells (HuC/D, magenta) and Müller glia (eGFP transgene, yellow) at different, crucial timepoints of retinal development (2, 2.5, 3 and 5 dpf) showing retinal progenitors in A–A‴. B–B‴ shows nascent IPL and Müller glia formation. In C–C‴ and D–D‴, IPL sublamination and Müller glia elaboration is evident. IPL, inner plexiform layer. All images representative of three experimental repeats. Scale bars: 25 µm (whole retina); 10 µm (magnified images).

Zebrafish photoreceptors undergo rapid development, with light-sensitive opsin mRNA expression detectable by 60 hpf (Robinson et al., 1995). Zebrafish are tetrachromats; they have rods and four cone photoreceptor subtypes, maximally sensitive to ultraviolet (UV), blue, green and red light. Different photoreceptor types are identifiable by specific markers; however, traditional methods make it challenging to label all photoreceptor subtypes such that they are distinguishable from one another. We combined antibody labelling with a transgenic line with fluorescently labelled rods [Tg(*rho:YFP*) line] to label all photoreceptors with subtype resolution in the developing zebrafish retina at three stages (3, 4 and 5 dpf) (Fig. 7). Of note, YFP signal could not be inactivated through LiBH_4_, light exposure or antigen retrieval, and therefore antibody labelling rounds were adjusted to avoid use of 488 fluorophores. We distinguished between the cone subtypes by utilising antibodies against UV, blue and red opsin (via 1D4), as well as arrestin 3a (with zpr-1). Zpr-1 labels both red and green cones; green cones can therefore be identified as cells that are arrestin 3a-positive but do not stain for red opsin (Fig. S6). At 3 dpf, developing cone photoreceptors stain with GNAT2 and zpr-1 (Fig. S5A′′), and have small outer segments labelled with antibodies for PRPH2, UV opsin, blue opsin and red opsin (Fig. S5A‴,A″″). Most of the cones with discernible outer segments were observed in the central retina. A few newly developed YFP-positive rods can also be observed in the retinal periphery. By 4 dpf, cones appear more morphologically mature with lengthened outer segments (Fig. S5B–B⁗). As mentioned, zebrafish cones are functional by 5 dpf and the animals begin to perform complex visually mediated behaviours, such as prey capture (Patterson et al., 2013). Corresponding with this, 5 dpf zebrafish cones have visually longer outer segments compared to 4 dpf with a tapered morphology (Fig. S5C–C⁗). Furthermore, there appear to be phagosomes in the RPE staining for zpr-1, GNAT2, UV opsin and PRPH2, suggesting that there is outer segment disc shedding at this stage.

During development, nascent MG cells begin as simple unbranched radial cells at 2.5 dpf, before morphologically elaborating to a mature, highly branched structure by 5 dpf (Williams et al., 2010; MacDonald et al., 2015; Wang et al., 2017). MG are among the last retinal cell types to mature, integrating into neuronal circuits when neurons are undergoing robust synaptogenesis (Cepko et al., 1996). To determine MG specification relative to development of other retinal neurons and inner plexiform layer (IPL) formation, we used markers for MG, namely, Zrf-1 (recognising Gfap), GS and the Tg(*Tp1:EGFP-CAAX*) transgenic reporter line, which labels retinal progenitors and MG (MacDonald et al., 2015; Kugler et al., 2023). We labelled amacrine cells and retinal ganglion cells with HuC and HuD (hereafter HuC/D), horizontal cells with CA-1, bipolar cells with PKC-β and synapse formation with Ribeye-A. We observed retinal progenitors at 2 dpf (Fig. 7A–A‴) labelled by the GFP transgene, corresponding to retinal ganglion cell (RGC) specification below the IPL, before MG genesis and onset of MG cell body basal migration at 2.5 dpf. MG are labelled by the transgene and Zrf1 at 2.5 dpf, but GS labelling is not yet apparent (Fig. 7B–B‴). At this point, the nascent IPL is present, as evidenced by the separation of the HuC/D signal and presence of ribbon synapses (Ribeye-A) (Fig. 7B′,B‴). From this timepoint, horizontal cells are visible, marked by carbonic anhydrase (CA) below the outer plexiform layer (Fig. 7B′,C′,D′). At 3 dpf, IPL expansion and bipolar cell terminal stratification is seen (Ribeye-A and PKC-β), along with the beginning of organisation of the IPL into clear sub-laminae (Fig. 7C′–C‴). Additionally, MG cell bodies have migrated to their final positions (Fig. 7C″) and are marked by GS labelling. By 5 dpf, the ribbons are organised into discrete layers in the IPL, with a visible separation between the ON and OFF layers and MG have elaborated processes into this layer to provide homeostatic support functions (Fig. 7D–D‴). As such, we used IBEX to describe retinal histogenesis, neuron migration and patterning relative to glial specification and morphogenesis across retinal development in the zebrafish. In conclusion, we were able to employ the IBEX technique and specifically designed antibody panels to describe multiple cell types at key stages of retinal development and explore their cellular relationships not possible with traditional methods.

### IBEX on frogs and killifish

**Fig. 8.**
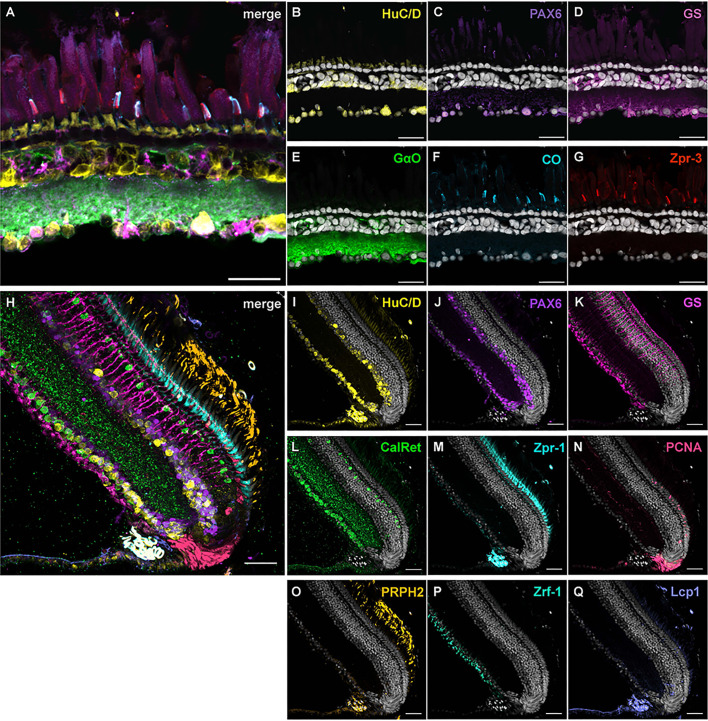
**IBEX on African clawed frog and turquoise killifish retina.** (A–G) 5-month-old *Xenopus laevis* tadpole sagittal retinal sections labelled with HuC/D (B, yellow), PAX6 (C, purple), GS (D, magenta), GαO (E, green), cone opsin (F, cyan) and zpr-3 (G, red). HuC/D labelled cells within the GCL and amacrine cell sublayer, as seen in zebrafish, as well as photoreceptor inner segments. PAX6 labelled primarly cells within the GCL, potentially RGCs and/or displaced amacrine cells. Müller glia were labelled with GS. GαO labelled bipolar cell bodies and processes within the INL and IPL, respectively. L and M cone outer segments were co-labelled with CO and zpr-3. (H–Q) 8-week-old killifish retinal sections labelled with HuC/D (I, yellow), PAX6 (J, purple), GS (K, magenta), Calretinin (L, green), Zpr-1 (M, cyan), PCNA (N, red), PRPH2 (O, orange), Zrf-1 (P, light green) and Lcp1 (Q, lavender/light purple). Huc/D and PAX6 labelled cells within the GCL and amacrine cell sublayer, as seen in zebrafish and frogs. GS and zrf-1 labelled Müller glia. Calretinin labelled cells within the GCL and amacrine cell sublayer. Zpr-1 labelled entire double cone cells, and PRPH2 labelled photoreceptor outer segments. PCNA expression was observed in the CMZ and in the photoreceptor cell layer. Lcp1 was observed in the INL, close to the CMZ, and within the photoreceptor cell layer. GCL, ganglion cell layer; INL, inner nuclear layer; IPL, inner plexiform layer; RGC, retinal ganglion cell. All images representative of three experimental repeats Scale bars: 25 µm (*Xenopus*, A–G); 30 µm (killifish; H–Q).

To verify the versatility of the IBEX technique across various animal models, we conducted IBEX on retinas of *Xenopus laevis* tadpoles and adult turquoise killifish. We performed IBEX on *Xenopus laevis* tadpole retinas to label different retinal cell types using antibodies against HuC/D, PAX6, GS, GαO, cone opsin (CO) and zpr-3 (Fig. 8A–G; Movie 3). HuC/D labelled cells within the INL and GCL, as well as photoreceptor inner segments. PAX6 labelled cells within the GCL – either displaced amacrine cells or RGCs – whereas GS labelled Müller glia. GαO labelled bipolar cell bodies and densely labelled processes within the IPL. L and M cone outer segments were labelled with both CO and zpr-3. The anti-CO antibody was raised against red opsin (lws), whereas zpr-3 is an anti-rhodopsin antibody that labels the green cone opsin as well as rod rhodopsin in zebrafish; however, in frogs it does not appear to label rod cells. IBEX was further conducted on the retinas of 8-week-old male African turquoise killifish (Fig. 8H–Q; Movie 4). Calretinin immunoreactivity was observed in ganglion and horizontal cells. HuC/D and PAX6 labelled amacrine and ganglion cells, and partial overlap of two markers was observed. Zpr-1 labelled entire double cone cells, whereas PRPH2 expression was seen in photoreceptor outer segments. Both GS and Zrf-1 (recognising Gfap) labelled MG cells. PCNA serves as an endogenous histologic marker for the G1/S phases of the cell cycle; therefore, PCNA expression serves as a marker of cell proliferation. In killifish retina, PCNA was densely expressed in the ciliary marginal zone (CMZ) and some signal was also found in the photoreceptor cell layer. The pan-leukocyte marker Lcp-1 was observed in the INL, close to the CMZ, and within the photoreceptor cell layer. As the eye ages, the number of Lcp-1-positive cells in the killifish retina is known to increase (Vanhunsel et al., 2021; Bergmans et al., 2024), hence explaining the sparse appearance of Lcp-1 staining in 8-week-old (young adult) retina shown in the figure.

## DISCUSSION

There is a lack of tools available to discriminate between more than three to four cell types in a tissue simultaneously. Here, we adapted the IBEX technique to vertebrate tissues and optimised the methodology to be compatible with zebrafish cell-specific fluorescent reporter lines, WMIF and *in situ* HCR. Furthermore, we employed IBEX to describe the relationships between glial cells and neurons and explore the entire complement of photoreceptor subtypes in the developing zebrafish retina. Additionally, we utilised IBEX to characterise the retina in turquoise killifish and *Xenopus laevis.* Therefore, IBEX is a robust method to multiplex markers and characterise cellular and molecular processes in the vertebrate model organisms.

### IBEX complements the existing zebrafish toolkit

We show that it is possible to combine IBEX with available transgenic lines with fluorescent protein expression. Zebrafish have a wealth of cell-specific transgenic reporter lines that drive transgene expression in each population of retinal cell. These have been valuable tools to characterise the development and degeneration of retinal cell types in many studies (Fadool, 2003; Bernardos and Raymond, 2006; Zolessi et al., 2006; Kimura et al., 2008; Vitorino et al., 2009; Almeida et al., 2014). We found that different fluorescent proteins have different fluorophore inactivation success, with GFP quenching reliably, and RFP and YFP not visibly quenching after treatment. BFP or CFP were not assessed. Thus, it is possible to pair IBEX with transgenic lines, although this should be tested on a case-by-case basis to determine whether endogenous fluorescent proteins can be inactivated, and subsequent labelling rounds adjusted accordingly.

Antibodies specific for a cell type or protein of interest can be limited in non-mammalian species, and this is especially true for poorly studied or newly discovered genes. Furthermore, studies might be interested in assessing spatial localisation of noncoding RNAs within tissues. As an alternative to antibody labelling, *in situ* hybridisation chain reaction (HCR) is a robust method to label mRNA of interest (Choi et al., 2010, 2016, 2018). We novelly combined *in situ* HCR with IBEX to spatially localise transcript expression for three genes with cellular markers across two cell types (three antibody labels). This can be applied to other systems to determine with high resolution which cell populations are expressing transcript for a gene of interest, by utilising the morphological information provided by antibody labelling. Hence, combining *in situ* HCR with IBEX is a powerful technique to identify expression patterns of genes of interest by localising gene expression to different immunolabelled cell types. Based on our results, IBEX complements multiplex HCR techniques, such as spectral unmixing (Schulte et al., 2024), to greatly enhance the number of proteins and mRNAs labelled on a single tissue.

WMIF is an effective technique to study the expression pattern of proteins while preserving the 3D structure of the tissue. This provides a powerful advantage over using tissue sections to gather spatial information, and has been used successfully in mouse, *Drosophila*, *Xenopus* and zebrafish tissues to identify patterns of protein expression. IBEX allows us to conduct multiple rounds of WMIF, which, to our knowledge, is the first reported instance of sequential whole-mount immunolabelling of the eye. This technique greatly increases the amount of information we can obtain from a single tissue and is a useful extension of WMIF to simultaneously visualise multiple proteins in their native location in tissues and study the spatial relationships between different cell types in whole tissues. Owing to the challenges of aligning samples similarly across rounds of immunolabelling and bleaching, it would be ideal to perform all the steps while the sample remains embedded in agarose, such as in a microfluidics setup.

### Potential of IBEX and multiplexing in other tissues and species

IBEX can be applied to study development and degeneration in various organs. It could be used in conjunction with cell death markers like Caspase-3 or TUNEL dyes to localise cell death or degeneration to specific cell types. Zebrafish are also a powerful model for regeneration research (Gemberling et al., 2013), and the ability to visualise multiple markers simultaneously can be applied to identify cells involved in regeneration and wound healing. While we have limited our study to zebrafish developmental stages, we anticipate IBEX with the markers used here will translate well to the adult tissues for the characterisation of ageing, degeneration and regeneration phenotypes. Finally, methods for rapid genetic screening have recently been developed in zebrafish using multiple guide RNAs to induce high rates of biallelic knockout of the gene of interest in injected embryos (crispants) (Kroll et al., 2020). IBEX can be utilised to efficiently characterise phenotypes in these crispants, which are often limited in number by injection capacity and efficiency.

IBEX can also be applied to any species where multiple cell-specific antibodies, fluorescent reporter transgenes and/or fluorescent *in situ* hybridisation techniques are available. However, these techniques and antibody panels will need to be validated on a case-by-case basis. These methodologies, some developed here, will be especially valuable where multiple different markers are required to confirm the identity of a cell, such as in immunological studies. We also demonstrate the benefits of IBEX for the characterisation of multiple cell types in unconventional model organisms, such as the killifish and *Xenopus laevis,* in which there are few transgenic reporter lines. The techniques demonstrated here can also be utilised for comparative anatomy studies, to label multiple cell types or subtypes in a tissue of interest before comparison between species. IBEX allows us to maximise the information that can be obtained from a single piece of tissue, a huge benefit for study of rare species or tissue that is difficult to access. Furthermore, it can be used to potentially identify subpopulations that would not be possible with conventional IF in such organisms. Labelling multiple markers on the same tissue will also have important implications for animal ethics and 3Rs (‘Replacement, Reduction and Refinement’) initiatives as the number of animals required for statistically significant phenotypic data is greatly reduced. Performing multiple rounds of IF on the same tissue produces rich datasets where the interactions between multiple cell types can be explored. As such, multiplexing techniques are not only powerful for data collection and exploring cellular relationships in whole tissues but also critical for efforts to reduce animal numbers in experiments.

### Antibody panel design and considerations

To maximise the potential of IBEX, we developed panels to label as many cell types as possible in each round of IBEX. Careful planning is required to design panels of markers (antibodies and lectins) to be used based on previous individual reactions and bleaching tests. The brightest and least efficiently bleached markers, for instance lectins or transgenes, were used in later or, ideally, as the last round to minimise the potential for significant leftover signal. Similarly, the weakest markers were used in early panels to increase the likelihood of strong signal detection. It is important to note that certain fluorophores are more amenable to bleaching than others, and later rounds are more susceptible to autofluorescence, particularly in the 555 channel as seen in Fig. 4 and Fig. S5, where some residual inner retinal staining is observed while labelling with GNAT-2. We ensured that the fluorophores which have been validated to bleach in the original protocol (Radtke et al., 2020) were used and any new fluorophores, such as the CoraLite^®^ Plus, were tested for bleaching prior to use in IBEX (Fig. 3F). It is possible to use secondary antibodies in IBEX, and this might be required for antibodies that fail to efficiently label via micro-conjugation. However, these secondaries must be incorporated into the first round of the IBEX if recognising a species from which multiple antibodies within the designed panel are raised, as subsequent rounds using antibodies raised in that same species will lead to cross-reactivity; in the case that there is a single antibody from a specific species within the panel, the antibody can be incorporated into any round using secondary antibodies. For most fluorophores, incubation with LiBH_4_ before washing was sufficient for bleaching the fluorophore. However, in some cases it required an antigen retrieval step (i.e. heating in sodium citrate), which efficiently quenches fluorophores. Importantly, after the antigen retrieval and IBEX procedure on zebrafish retinal tissue, the nuclei appeared qualitatively similar across three rounds of IF and imaging (Fig. S2) and were easily aligned using the SimpleITK registration software.

### Limitations and future work

There are some limitations to the IBEX approach. Micro-conjugations can be unreliable on occasion (i.e. not all antibodies successfully conjugate), there is a need for a relatively large volume of validated primary antibody (although much less than when performing a primary conjugation), and the technique works most effectively when the protein in question is highly abundant. Hence, when used for proteins that have low expression or low affinity for antibodies, the concentrations might have to be increased over traditional methods. Many antibodies do not have their exact antigen identified or validated in non-mammalian systems, such as zebrafish. However, as we were only looking at broad cell type distributions, rather than targeted molecular events, validation of each antigen was not necessary for this study. When possible, we did utilise antibodies with known or validated antigens. When using unvalidated antibodies, we selected those that had conserved labelling patterns and included other methods – such as transgenic lines and *in situ* hybridisation – to complement the labelling and provide information about specificity. Transgenic lines with RFP or YFP that are not inactivated [such as the Tg(*rho:YFP*) line used for the developmental series in Fig. 7] can still be used if required but would reduce the number of channels available for successive imaging rounds. Finally, there are likely limitations on the number of immunolabelling rounds that can be performed without tissue damage or visible residual signal from previous rounds. However, we have conducted four rounds of IBEX on retinal cryosections and there has been no noticeable degradation of tissue integrity or signal loss. As such, it may be possible to use >20 markers on a single zebrafish cryosectioned tissue, as there have been 20 rounds and 66 antibodies reported in human lymph nodes (Radtke et al., 2020).

The IBEX technique is compatible with standard confocal as well as epifluorescence microscopy. Here, we principally used a standard confocal microscope with four excitation laser wavelengths (405, 488, 555 and 647 nm), which allowed a maximum of three antibodies plus DAPI in each panel. However, this can be expanded upon if your microscope has additional spectral capabilities (e.g. tuneable or white light laser) and antibodies are visualised with additional fluorophores via conjugation or secondary antibodies. To this end, we also conducted IBEX using an epifluorescence microscope with *z*-stack capabilities and expanded spectral excitation properties to visualise ten markers on the same tissue (Fig. S3). As such, this technique will be applicable to any fluorescence microscopes available where multiple channels can be acquired on the same tissue.

### Conclusions

In conclusion, we have adapted a highly multiplexed immunofluorescence technique (IBEX) to visualise many targets in a single zebrafish retina sample. We have established a pipeline that can be used to carry out multiplexed imaging on multiple zebrafish tissue samples at different timepoints at once. We successfully modified the IBEX protocol to be compatible with *in situ* HCR as well as whole-mount tissue and characterised cellular relationships in the developing retina across every major cell type in the tissue. We further applied this technique to label several cell types in the retina of unconventional model organisms with scarcity of tissue, namely, killifish and *Xenopus laevis.* Therefore, this technique can be a powerful method to explore multicellular tissues in zebrafish, and other model organisms.

## MATERIALS AND METHODS

### Animals

Adult fish were housed in the animal facility at the University College London on a 14-h-light–10-h-dark cycle at 28°C, following previously established husbandry protocols (Westerfield, 1993). Experimental procedures were conducted in accordance with the UK Home Office Animals (Scientific Procedures) Act 1986 (zebrafish project license PPL: PP2133797, held by R.B.M, and killifish PPL: PP7179495 held by Dr Elspeth Payne). Zebrafish embryos were obtained by light-induced spawning, collected in E3 buffer (5 mM NaCl, 0.17 mM KCl, 0.33 mM CaCl2, 0.33 mM MgSO4) with or without Methylene Blue, and maintained in an incubator at 28.5°C until use.

*Xenopus laevis* (African clawed frog) use was approved by the University of Alberta Animal Use and Care Committee (AUP00004203) and carried out in accordance with the Canadian Council on Animal Care. Frogs were housed at 18°C under a 12-h cyclic light schedule (7:00–19:00; 900–1200 lux).

### Animal strains

Wild-type zebrafish embryos (ABTL/Tübingen) were used for the adaptation of the IBEX technique on section IF, WMIF, and combined HCR and IF. Tg(*GFAP:GFP*) (Bernardos and Raymond, 2006), Tg(*vsx1:GFP*)^nns5^ (Kimura et al., 2008), Tg(*TP1:Venus-Pest*) (Ninov et al., 2012), Tg(*tp1bglob:eGFP-CAAX*) (Kugler et al., 2023) and Tg(*ptf1a:dsRed*)^ia6^ (Jusuf et al., 2012) were used to optimise the protocol for IBEX using transgenics. Tg(*rho:YFP*)^gm500^ (White et al., 2017), Tg(*tp1bglob:eGFP-CAAX*) embryos were used at different timepoints to study development of neurons and glia. Wild-type killifish adults (GRZ) and wild type *Xenopus laevis* were used for adaptation of IBEX on retinal sections.

### Preparation of retinal sections

Animals at the desired stages were overdosed with 0.4% tricaine and fixed in 4% paraformaldehyde overnight at 4°C. Tissues were washed three times for 5 min in PBS and then immersed in either 20% (frogs) or 30% (fishes) sucrose in PBS and allowed to sink overnight. Samples were embedded in OCT (Sigma-Aldrich, cat. no. SHH0024) and frozen at −80°C. Frog eyes were obtained by B.J.C. Froglets aged 145 days post-fertilization were euthanised by overdose with tricaine (0.5% until unresponsive to toe pinch), decapitation and then pithing. Whole eyes were fixed in 4% PFA plus 3% sucrose overnight, cryoprotected in 20% sucrose overnight with gentle shaking, and then shipped to UCL in 20% sucrose for further processing. SuperFrost™ Plus Adhesion Microscope Slides (Epredia, cat. no. J1800AMNZ) were coated evenly with 5 μl chrome alum gelatin (Newcomer Supply, part #1033C) and dried in an incubator at 60°C for 1 h, to minimise loss of tissue over multiple rounds of immunolabelling. Retinal sections of embedded tissues were sectioned onto these slides at a thickness of 12–14 µm using a cryostat (Leica CM1950) and left at room temperature (RT) to dry overnight. Slides were then stored at −80°C.

### Antibody micro-conjugation

All antibody and FlexAble details are found in Table S1. Each antibody was tested with the FlexAble micro-conjugation kits at the recommended concentration (0.5 µg) per slide. However, this did not label cells efficiently in retinal sections. Doubling the concentration of primary antibody was found to stain tissue more effectively. 1 µg of each primary antibody was combined with 2 µl of FlexAble linker protein for the desired fluorophore, and the volume was made up to 16 µl with the provided buffer, following kit recommendations. This solution was incubated for 5 min in the dark at RT, and then 4 µl of quencher solution was added and left to incubate in the dark at RT for a further 5 min, according to the manufacturer's protocol. The entire reaction volume for each antibody was used for subsequent steps.

### IBEX technique

This method is an adapted version of the original IBEX protocol (Radtke et al., 2020) and an overview is given in Fig. 2. The sections were rehydrated in PBS for 5 min at RT. Antigen retrieval was then performed by heating the slides for 20 min in 10 mM sodium citrate (pH 6) in a rice cooker at ∼100°C. Antigen retrieval was performed on all tissues used in this study prior to the first round of antibody incubation, including primary or directly conjugated antibodies. The sections were blocked for 1 h in block solution (10% goat serum, 1% BSA, 0.8% Triton X, 0.1% Tween 20, made up with PBS) at RT. Primary antibodies were conjugated to fluorophores as described above. The slides were incubated with the first round of antibodies, diluted appropriately in block solution, at 4°C overnight. Following three 20-min washes with PBS, secondary antibodies were added, if needed. Slides were then incubated at RT for 2 h or overnight in 4°C and washed after with PBS three times for 10 min. Slides were mounted in Fluoromount G mounting medium (cat. no. 00-4958-02, Invitrogen) and imaged on a Leica THUNDER imager, Leica SP8 confocal microscope or Zeiss LSM 900 inverted confocal using four or five channels (405, 488, 550, 647 and 750 nm).

After image acquisition, slides were placed in a 50 ml falcon tube filled with PBS and left until the coverslip fell off, and then washed three times to remove the mounting medium. Fluorophores were bleached by incubating slides in 150 µl of LiBH_4_ (16949-15-8, STREM) solution (1-2 mg/ml) under direct white light (minimum of 300 lux) for 30 min. The slide was then washed three times for 10 min in PBS before the next round of antibodies was added and steps were repeated as above.

### IBEX for combined *in situ* HCR/IF on sections

HCR probes for *cyp26a1* and *vsx1* were kindly gifted by Takeshi Yoshimatsu, while the probe set for *glula* was designed using a custom script (Trivedi and Powell, unpublished) and ordered from Life Technologies, Thermo Fisher Scientific. HCR amplifiers (Alexa Fluor 488, Alexa Fluor 546 and Alexa Fluor 647), and buffers (hybridisation, wash, and amplification) were purchased from Molecular Instruments (https://www.molecularinstruments.com/). A published *in situ* HCR protocol (Choi et al., 2018) was adapted for retinal zebrafish sections. Slides were rehydrated in PBS for 5 min and then treated with 250 µl proteinase K (20 µg/ml) for 10 min at RT. They were washed twice with PBS plus 0.1% Tween 20 (PBST) and post-fixed with 250 µl of 4% paraformaldehyde for 20 min at RT. Slides were washed three times for 5 min with PBST and incubated in 150 µl of probe hybridisation buffer at 37°C for 30 min. Sections were then incubated overnight at 37°C in probe solution (4 µl of each probe set, made up to 150 µl in probe hybridisation buffer). Excess probes were removed by washing slides four times for 15 min in probe wash buffer at 37°C, and then two times for 5 min with 5× SSCT buffer (cat. no. 15557044, Thermo Fisher Scientific; 20× SSC diluted to 5× concentration with distilled water, and 0.1 % Tween 20 was added) at RT. Slides were pre-amplified in 150 µl of amplification buffer for 30 min at RT. 4 µl each of hairpin 1 and hairpin 2 amplifier were snap cooled by heating to 95°C for 90 s and cooling to RT. These were then added to the slides in 150 µl of amplification buffer and incubated overnight at RT in the dark. Slides were washed four times for 5 min in 5× SSCT buffer and mounted in Fluoromount G medium. Sections were imaged on the Zeiss LSM 900 with a 40× immersion oil objective (Na 1.1) using four channels (405, 488, 546 and 647 nm). After image acquisition, slides were heated to 60°C in sodium citrate (pH 6) for 20 min to quench fluorophores. IF was then repeated on sections as described above.

### IBEX for WMIF

Wild-type zebrafish embryos were treated with 0.0045% phenylthiourea from 6 hpf to prevent pigment formation and at 5 dpf, were overdosed with 0.4% tricaine and fixed in 4% paraformaldehyde overnight at 4°C. They were washed in PBST and heated in 10 mM sodium citrate (pH 6) at 70°C for 15 min. Samples were washed twice for 10 min in PBST, twice for 5 min in distilled water and incubated with ice-cold acetone for 20 min at −20°C. This was followed by three 5 min washes in PBS and incubation in blocking solution (10% goat serum, 0.8% Triton X-100, 1% BSA in PBST) for 2 h at RT. Micro-conjugation of the antibodies was carried out as described above, doubling the volume of antibody used for sections. Embryos were incubated in antibody solution with DAPI, diluted with blocking solution, at RT overnight with gentle agitation at room temperature. After three 1-h washes in PBS plus 1% Tween 20, embryos were mounted in molten 1% low-melting-point agarose in PBS, in a glass bottomed dish. Once hardened, they were covered with 1× PBS and imaged on the Zeiss LSM 900 with a 40× immersion oil objective (Na 1.1) using four channels (405, 488, 546 and 647 nm). Embryos were then extracted from the agarose and fluorophore signal was inactivated with LiBH_4_ solution mentioned above in individual tubes for 2 h at room temperature without bright light. The antibody incubation process was repeated, keeping embryos separate in order to overlay correct images during registration. They were then carefully mounted in a manner that was as similar to that for the previous round as possible and imaged as above, keeping imaging parameters like stack size and step size consistent between rounds.

### Optimization of inactivation of fluorophores

Fluorophores react differently to bleaching with LiBH_4_ under bright light and therefore we used several different methods to inactivate fluorophore signal between rounds of labelling. CoraLite micro-conjugated antibodies bleached after 30 min of LiBH_4_ treatment under bright light. The same antibodies required 2 h of incubation in LiBH_4_ solution to observe reduction in signal in WMIF. In cases where an Alexa Fluor 555-conjugated secondary antibody was used, 30 min of heating at 60°C in sodium citrate solution (pH 6) in an oven was needed to quench the fluorescence. For transgenic lines, antigen retrieval by boiling slides in sodium citrate solution (pH 6) for 20 min inactivated the fluorescent protein, but when boosted with the anti-GFP primary and Alexa Fluor secondary antibodies, the signal was not reduced even after antigen retrieval. Hence, fluorophores must be individually tested to assess suitability for use with IBEX, and ones that do not show reduction in signal used in the last round of immunolabelling.

### Image processing and alignment

Imaging parameters such as stack size, number of steps, step size, scan speed and resolution were kept consistent across all rounds of imaging. Additionally, the first and last slices of the stacks in later rounds were set to match those of the first round of imaging as closely as possible, using DAPI as a visual reference. For whole-mount alignment, each *z*-section was aligned manually across IF rounds before maximum projection of the registered *z*-stack. Once all rounds were completed, Imaris (Oxford Instruments) was used to process the images. Brightness, contrast and colours were adjusted and filters, such as Gaussian, were applied where appropriate. This can also be conducted using freeware (e.g. Fiji). Once all images were processed, the SITK-IBEX registration code (Radtke et al., 2022) was used to register the *z*-stacks. This open-source Python code (https://github.com/niaid/sitk-ibex) can be used through an Imaris Xtension or as a standalone code. It requires a free image viewer, like Imaris or Aivia, to rename files in a format recognizable by the code for registration. Once channels in each file are named with a consistent identifier such as ‘Alexa-647’, the registration code aligns them based on the reference channel, in our case, the nuclear stain DAPI. Maximum projection images were then obtained using the snapshot feature while in 3D viewer or using Fiji.

## Supplementary Material



10.1242/joces.263407_sup1 Supplementary information
