# Supplementary material for: ‘Iterative Bleaching Extends Multiplexity’ facilitates simultaneous identification of all major retinal cell types
Source: J Cell Sci. 2024 Dec 10;137(23):jcs263407. doi: 10.1242/jcs.263407 (PMC11827602; doi:10.1242/jcs.263407)
Supplement: Supplementary information [file joces-137-263407-s1.pdf]

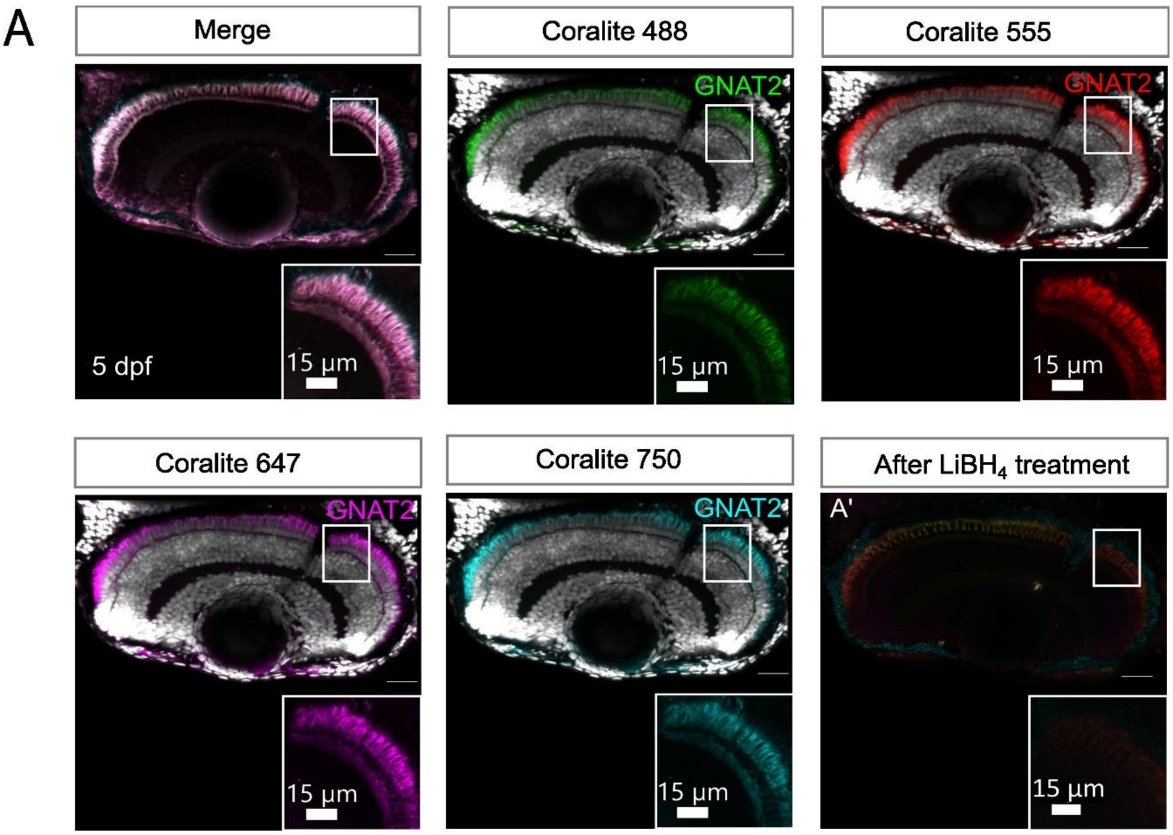

Fig. S1. (A) Epifluorescence images of cone photoreceptor cells immunolabelled with the GNAT2 antibody on the same single retinal section. Rabbit α-GNAT2 is conjugated to Coralite 488, Coralite 550, Coralite 647 and Coralite 750. (A′) Decreased signal after bleaching the sample with LiBH4 under bright light. Scale bars - 40µm for whole retina, 15µm for zoom images.

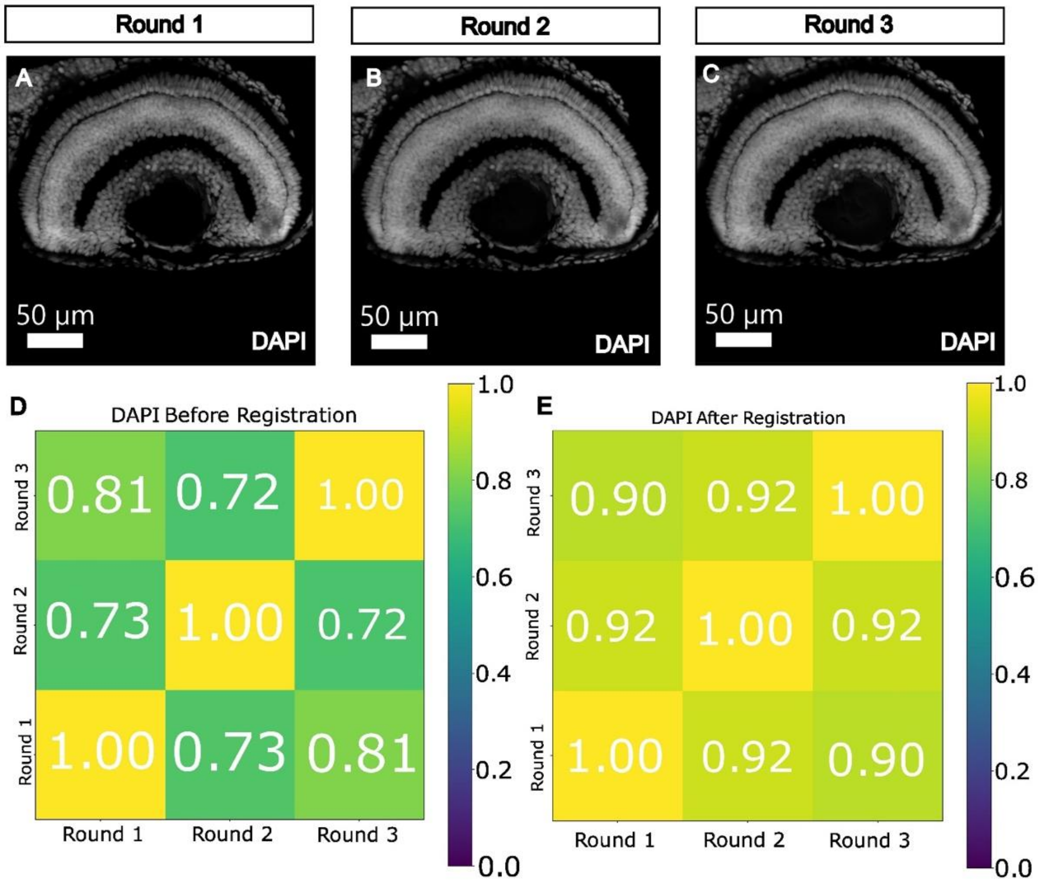

Fig. S2. Three round IBEX run on 5 dpf zebrafish retina cryosections with Z-stacks using a 63x objective and 2x optical zoom on the Leica SP8 confocal microscope (three separate technical repeats were performed). (A-C) Maximum projections of DAPI staining during the imaging of rounds 1-3, respectively. (D) Correlation matrix for DAPI staining across the rounds before Simple ITK registration of Z-stacks. (E) Correlation in DAPI staining across the rounds after affine registration of Z-stacks. Scale bars - 50µm.

Journal of Cell Science • Supplementary information

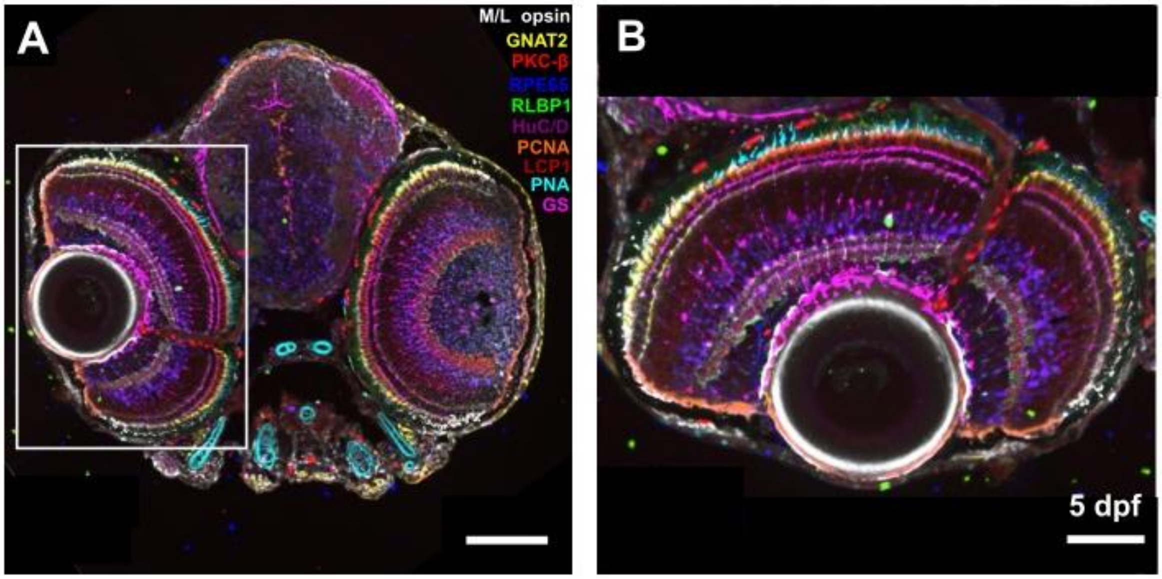

Fig. S3. Three round IBEX run using the Leica THUNDER imager with a 40x air objective following instant computational clearing.(A) Epifluorescence images of 5dpf zebrafish retinal section immunolabelled with a panel of 9 antibodies and a lectin stain: RPE65 (dark blue), GS (magenta), PKC-β (red), HuC/D (purple), PCNA (orange), GNAT2 (yellow), RLBP1 (green), Lcp-1 (maroon), M/L opsin (white) and PNA stain (cyan). (B) Zoom in of region of interest indicated in A, with merge of the 10 antibodies used. Scale bar - 80µm (A) and 40µm (B).

Journal of Cell Science • Supplementary information

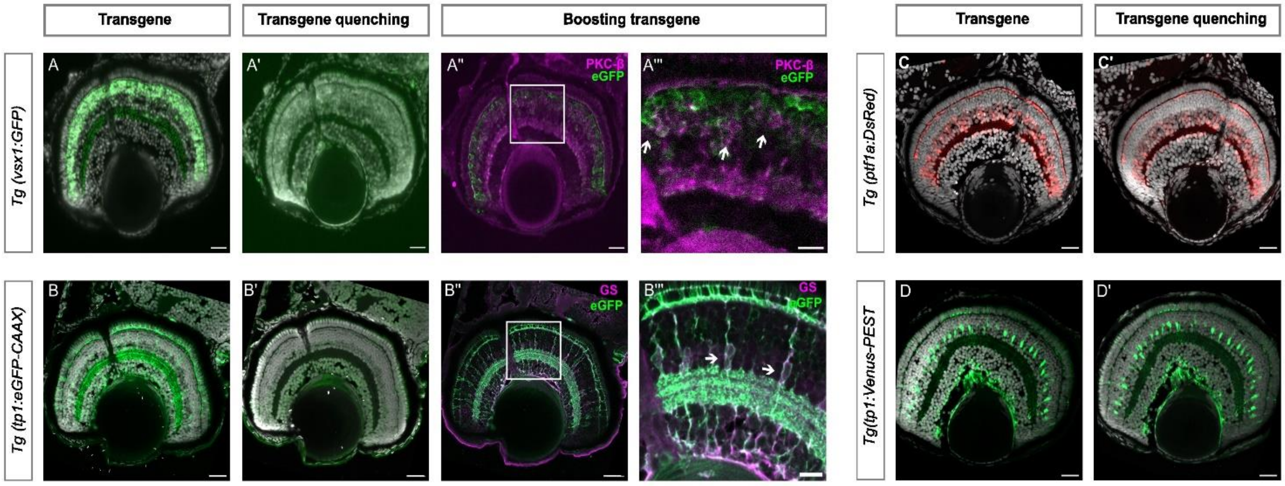

Fig. S4. IBEX is compatible with transgenic fluorescent reporter lines. (A) Images of 5 dpf cytosolic GFP (Tg(*vsx1:GFP*) transgenic labelling bipolar cells) positive retinal sections without any treatment. (A′) Images of the same section after sodium citrate treatment, showing reduced signal. (A″) Section imaged after boosting transgene with anti-GFP antibody and co-labelling with PKCβ to confirm specificity of boosted transgene. (A‴) Zoom in of region of interest in (A″). (B) Confocal images of 5 dpf membrane bound GFP (Tg(*tp1:eGFP-CAAX*) transgenic labelling MG membrane) positive retinal sections without any treatment. (B′) Images of the same section after sodium citrate treatment, showing reduced signal. (B″) Section imaged after boosting transgene with anti-GFP antibody and co-labelling with GS to confirm specificity of transgene (zoom: arrows). (B‴) Zoom of region of interest indicated in (B″). (C, D) Confocal images of RFP (Tg(*ptf1a:Dsred*) transgenic) and YFP (Tg(*tp1:Venus-PEST*) transgenic) positive retinal sections without treatment. (C′,D′) RFP and YFP transgenes do not show any change in signal after sodium citrate treatment. dpf: days post fertilisation, MG: Müller glia. Scale bar -25µm.

Journal of Cell Science • Supplementary information

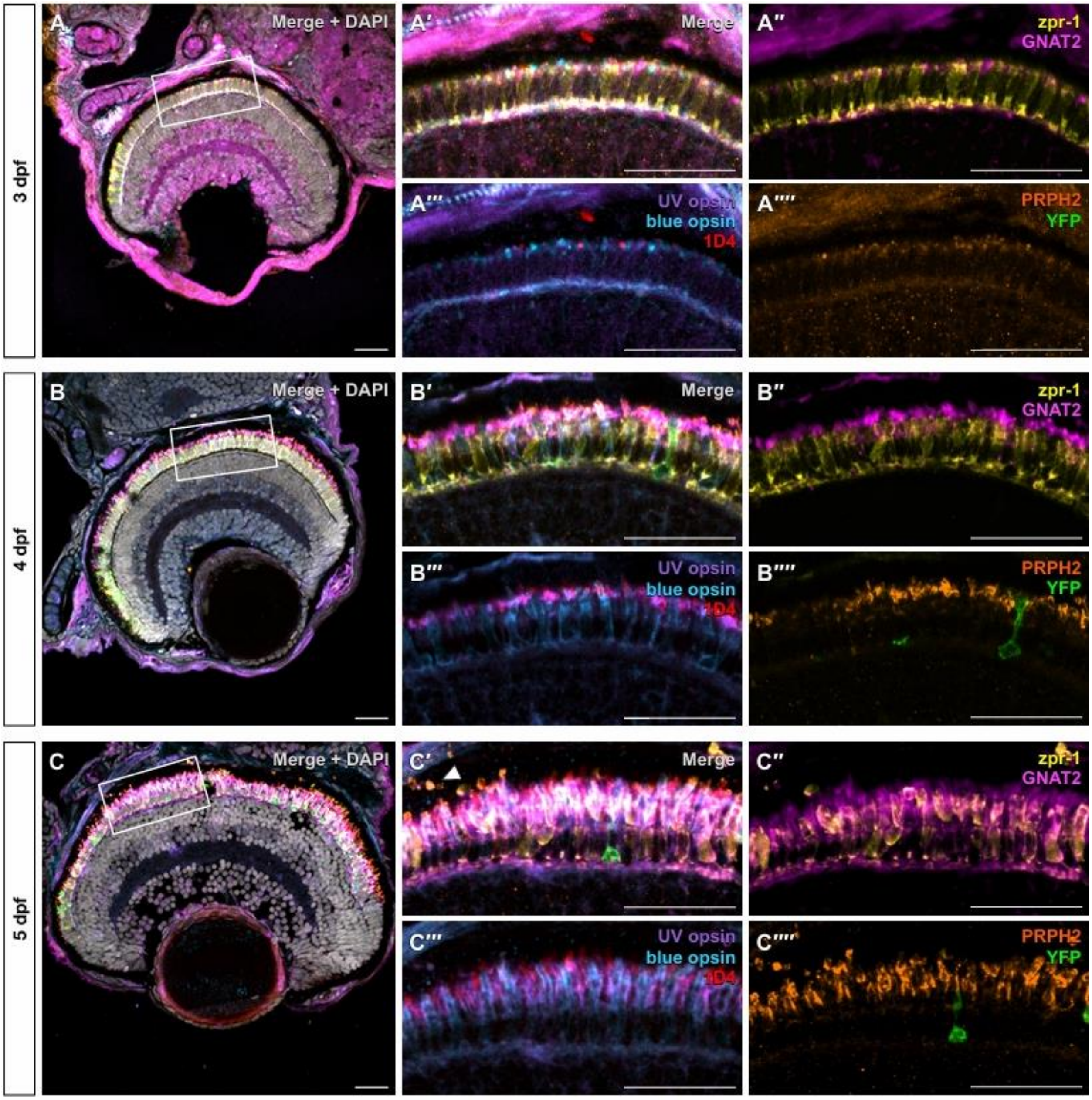

Fig. S5. Visualisation of all photoreceptor subtypes during zebrafish development using IBEX. Sagittal sections of Tg(*rho:YFP*) embryos labelled with zpr-1 (yellow), GNAT2 (pink), UV opsin (purple), blue opsin (blue), 1D4 (red), and PRPH2 (orange) at 3 (A), 4 (B), and 5 (C) dpf. YFP is shown in green. (A′, B′, C′) Show zooms of photoreceptors will all labels merged, without DAPI; (A″, B″, C″) shows zpr-1 and GNAT2 labelling; (A‴, B‴, C‴) shows UV, blue, and red opsin (1D4) labelling; and (A⁗, B⁗, C⁗) shows PRPH2 labelling and YFP. (A) 3 dpf retinas have small, newly developing outer segments visible by UV opsin, blue opsin, red opsin (1D4), and PRPH2 labelling. Entire cone cell bodies are visible by GNAT2 labelling, while red and green cone cell bodies are visible by arrestin 3a (zpr-1) labelling. Newly formed rods are visible at the periphery of the retina by YFP labelling. (B) 4 dpf embryos have longer outer segments with increased PRPH2 staining, indicative of disc structure. (C) 5 dpf embryos have long outer segments that are visibly starting to taper into more of a “cone-like” morphology. dpf: days post fertilisation. Scale bars - 25µm.

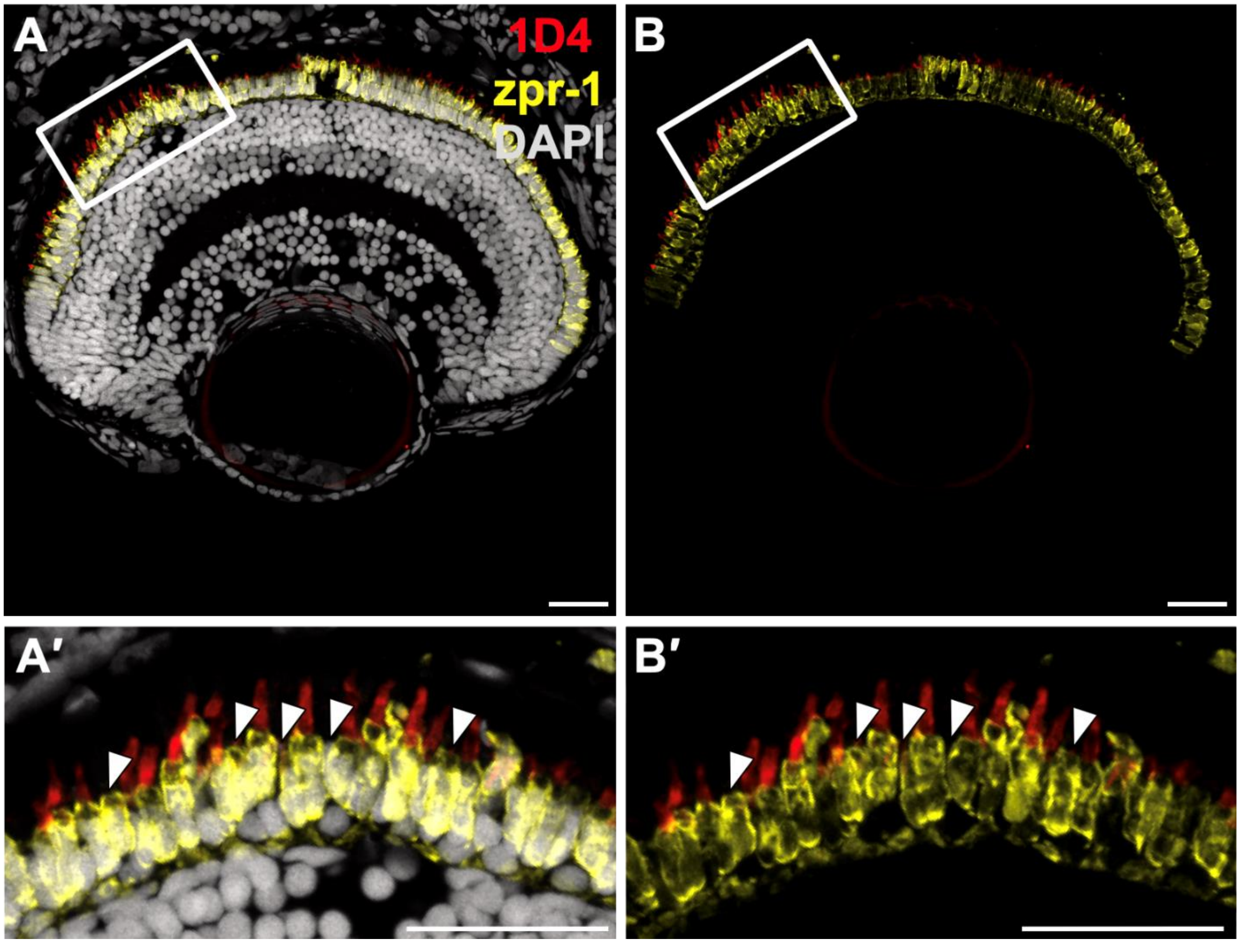

Fig. S6. Discrimination of green cones in 5 dpf zebrafish retina. Red cones are positive for both zpr-1 and 1D4 (red opsin), whereas green cones are positive for only zpr-1 (arrowheads). Scale bars - 25µm.

Journal of Cell Science • Supplementary information

Table S1. Antibodies and reagents used for immunofluorescence and IBEX.

Primary antibodies					
**REAGENT OR RESOURCE**	**CELLULAR TARGET IN ZEBRAFISH**	**HOST**	**SOURCE**	**IDENTIFIER**	**DILUTION**
α-1D4	Red cone Photoreceptors	Mouse monoclonal	Santa Cruz Biotechnology	Cat. No. sc-57432	1:50
α-ARR3	Cone photoreceptors	Rabbit polyclonal	Merck	Cat. No. AB15282	1:50
α-Blue opsin	Blue cones	Rabbit polyclonal	Kerafast	Cat. No. EJH012	1:50
α-Calbindin	Amacrine cell subpopulation, Retinal ganglion cells	Rabbit	Swant	Cat. No. CB38a	1:50
α-Calretinin	Amacrine cells subpopulation, Retinal Ganglion cells	Rabbit polyclonal	Swant	Cat. No. 7697	1:150
α-Carbonic anhydrase	Horizontal cells and Müller glia	Rabbit polyclonal	ABCAM	Cat. No. ab108367	1:50
α-cone opsin (lws, *Xenopus*)	N/A	Rabbit	N/A	N/A	1:200
α-GαO	N/A	Mouse	EMD Millipore	MAD3073	1:200
α-GFAP	Müller glia	Mouse	Biolegend	Cat. No. 837508	1:50
α-GFP	GFP	Chicken	Invitrogen	Cat: A11122	1:500

Journal of Cell Science • Supplementary information

α-GNAT2	Cone photoreceptors	Rabbit polyclonal	MBL	Cat. No. PM075	1:75
α-GS	Müller glia	Mouse	Proteintech	Cat. No. CL488-66323	1:50
α-HuC/D	Amacrine cells & Retinal Ganglion cells	Mouse	Invitrogen	Cat. No. A21271	1:100
α-LCP1	Macrophages	Rabbit polyclonal	Proteintech	Cat. No. 13025-1-AP	1:50
α-LCP1	Macrophages	Rabbit polyclonal	GeneTex	Cat. No. GTX124420	1:50
α-M/L opsin	Cone photoreceptors	Rabbit	Millipore Merck	Cat. No. AB5405	1:50
α-PAX6	Amacrine cells	Rabbit polyclonal	Proteintech	Cat. No.	1:50
α-PCNA	Proliferating cells	Rabbit polyclonal	Proteintech	Cat. No. 24036-1-AP	1:50
α-PCNA	Proliferating cells	Mouse monoclonal	Santa Cruz Biotechnology	Cat. No. SC-56	1:50
α-PKC-β	Rod bipolar cells and their terminals	Rabbit Polyclonal	Proteintech	Cat. No. 12919-1-AP	1:50
α-PRPH2	Photoreceptors	Rabbit polyclonal	Proteintech	Cat. No. 18109-1-AP	1:200
α-Ribeye-A	Bipolar cell ribbon synapses	Rabbit polyclonal	Gift from Teresa Nicholson	NA	1:500

Journal of Cell Science • Supplementary information

α-RLBP1	Müller glia	Rabbit polyclonal	Proteintech	Cat. No. 15356-1-AP	1:50
α-RPE65	Retinal pigment epithelium	Rabbit polyclonal	Proteintech	Cat. No. 17939-1-AP	1:25
α-UV opsin	UV cone photoreceptors	Rabbit polyclonal	Kerafast	Cat. No. EJH013	1:50
α-Zo1	Tight junctions	Mouse Monoclonal	Life technologies	Cat. No. 339100	1:150
α-Zpr1	Double cone photoreceptors	Mouse	ZIRC	ZDB-ATB-081002-43	1:200
α- Zpr-3	Green cone photoreceptors, rod photoreceptors	Mouse	ZIRC	ZDB-ATB-081002-45	1:200
α-Zrf-1	Müller glia basal end	Mouse Monoclonal	ZIRC	Cat. No. ZDB-ATB-081002-46	1:25
Secondary antibodies					
α-Rabbit Alexa Fluor™ 647		Goat	Invitrogen	Cat. No. A-21244	1:1000
α-Rabbit Alexa Fluor™ 546		Goat	Invitrogen	Cat. No. A-11035	1:1000
α-Mouse Alexa Fluor™ 546		Goat	Invitrogen	Cat. No. A-11030	1:1000

α-Mouse Alexa Fluor™ 647		Goat	Invitrogen	Cat. No. A-21235	1:1000
α-Chicken Alexa Fluor™ 488		Goat	Invitrogen	Cat. No. A-11039	1:1000
Other markers					
Lectin PNA			Invitrogen	Cat. No. L21409	1:1000
DAPI			Invitrogen	Cat. No. D1306	1:1000
Conjugation kits					
FlexAble CoraLite® 488 Antibody Labeling Kit for Rabbit IgG			Proteintech	Cat. No. KFA001	
FlexAble CoraLite® Plus 550 Antibody Labeling Kit for Rabbit IgG			Proteintech	Cat. No. KFA002	
FlexAble CoraLite® Plus 647 Antibody Labeling Kit			Proteintech	Cat. No. KFA003	

Journal of Cell Science • Supplementary information

for Rabbit IgG					
FlexAble CoraLite® Plus 750 Antibody Labeling Kit for Rabbit IgG			Proteintech	Cat. No. KFA004	
FlexAble CoraLite® Plus 550 Antibody Labeling Kit for Mouse IgG1			Proteintech	Cat. No. KFA022	
FlexAble CoraLite® Plus 647 Antibody Labeling Kit for Mouse IgG1			Proteintech	Cat. No. KFA023	
ReadiLink™ Rapid iFluor® 594 Antibody Labeling Kit			AAT	Cat. No. 1230-AAT	

Journal of Cell Science • Supplementary information

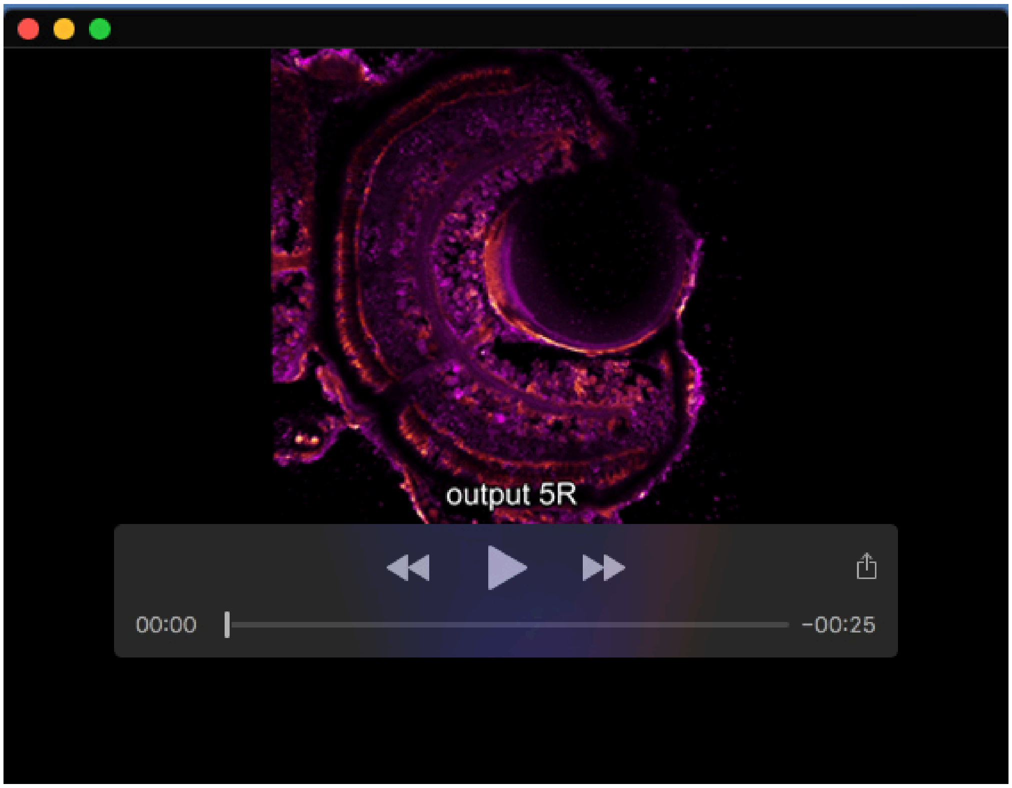

Movie 1. Maximum intensity projection of a registered, multiplexed 5dpf zebrafish retina in Fig. 4, displaying markers individually.

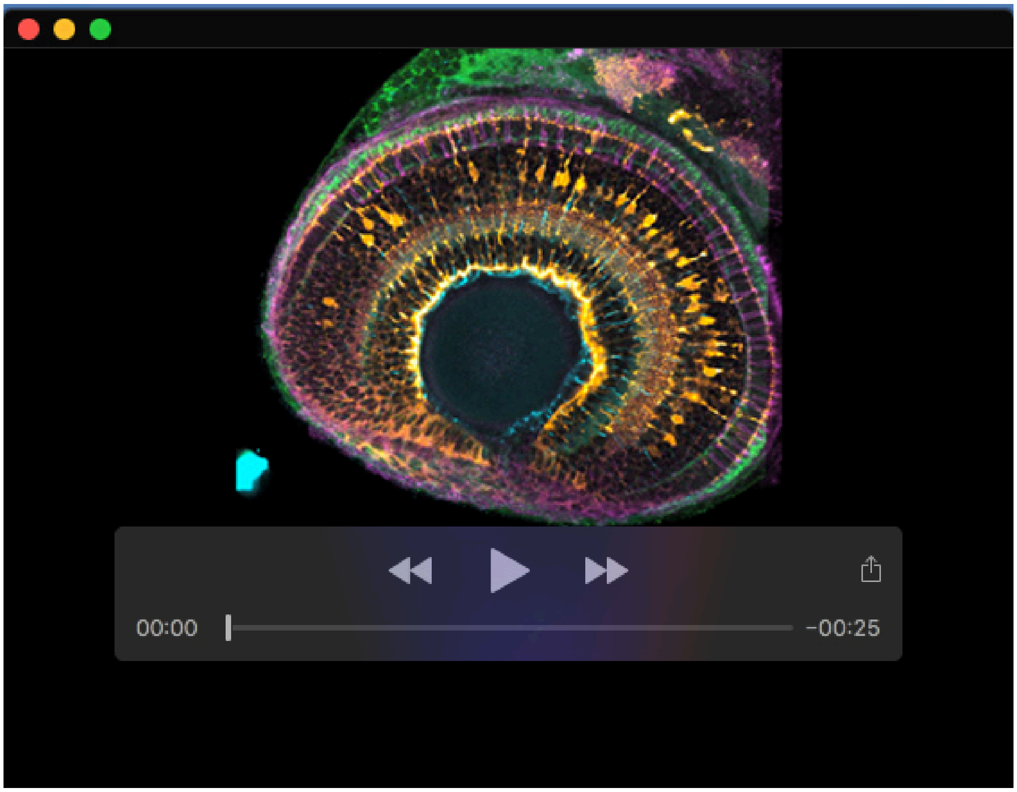

Movie 2. Movie showing the different optical planes in the z-stack of a multiplexed wholemount sample, registered to show 4 different markers, shown in Fig.6.

Journal of Cell Science • Supplementary information

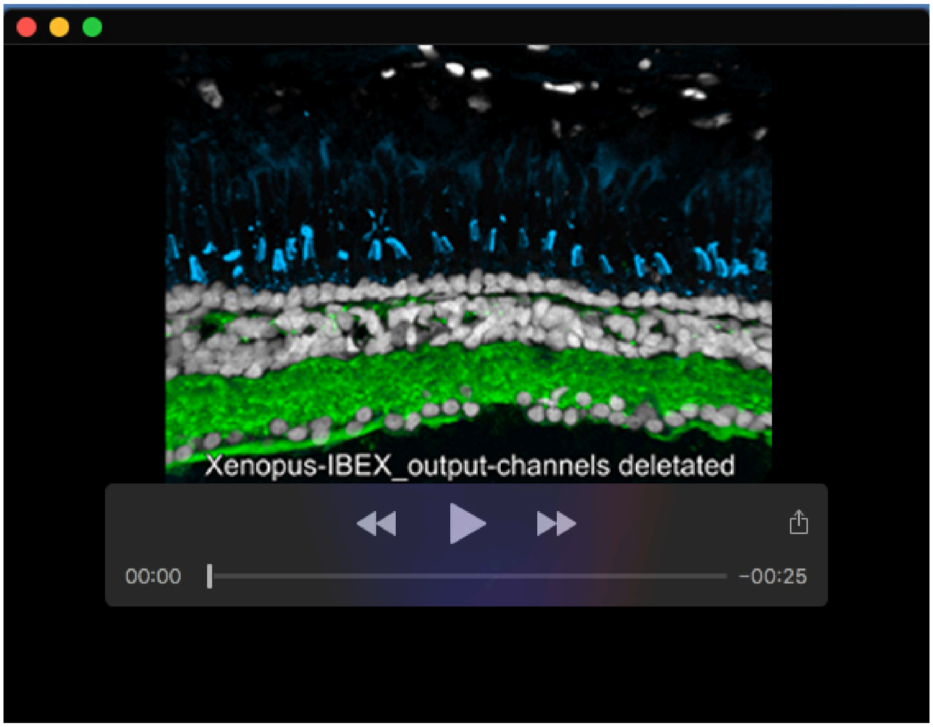

Movie 3. Maximum intensity projection of a registered, multiplexed 5 month old *Xenopus laevis* retina in Fig. 8, displaying markers individually.

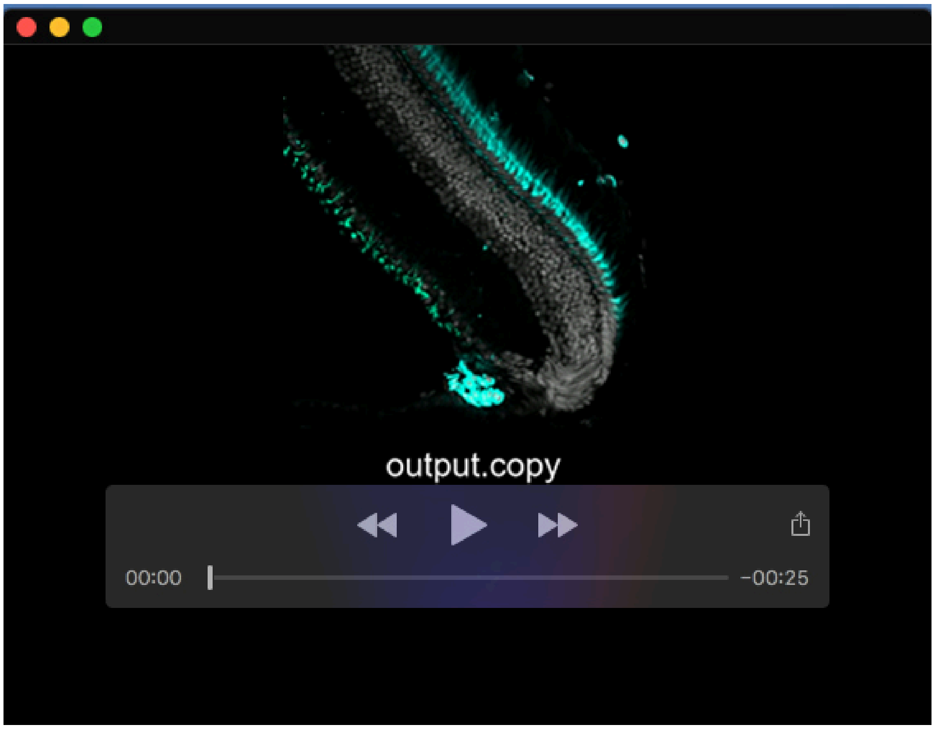

Movie 4. Maximum intensity projection of a registered, multiplexed 8 week old Killifish retina in Fig. 8, displaying markers individually.

Journal of Cell Science • Supplementary information
