# Supplementary material for: ‘Iterative Bleaching Extends Multiplexity’ facilitates simultaneous identification of all major retinal cell types
Source: J Cell Sci. 2024 Dec 10;137(23):jcs263407. doi: 10.1242/jcs.263407 (PMC11827602; doi:10.1242/jcs.263407)
Supplement: Supplementary file 1 [file joces-137-263407_review_history.pdf]

Journal of Cell Science

'Iterative Bleaching Extends Multiplexity' facilitates simultaneous identification of all major retinal cell types

Aanandita A. Kothurkar, Gregory S. Patient, Nicole C. L. Noel, Aleksandra M. Krzywańska, Brittany J. Carr, Colin J. Chu and Ryan B. MacDonald
DOI: 10.1242/jcs.263407

Editor: Caroline Hill

Review timeline
Original submission: 26 June 2024
Editorial decision: 29 July 2024
First revision received: 25 October 2024
Accepted: 3 November 2024

Original submission

<u>First decision letter</u>

MS ID#: jcs.263407

MS TITLE: Iterative Bleaching Extends Multiplexity (IBEX) imaging facilitates simultaneous identification of all cell types in the vertebrate retina

AUTHORS: Aanandita A. Kothurkar, Gregory S. Patient, Nicole C. L. Noel, Aleksandra M. Krzywańska, Brittany J. Carr, Colin J. Chu and Ryan B. MacDonald

We have now reached a decision on the above manuscript.
To see the reviewers' reports and a copy of this decision letter, please go to:
As you will see, the reviewers gave favourable reports but raised some critical points that will require amendments to your manuscript. I hope that you will be able to carry these out because I would like to be able to accept your paper, depending on further comments from reviewers.

I should be grateful if you would also provide a point-by-point response detailing how you have dealt with the points raised by the reviewers in the 'Response to Reviewers' box. Please attend to all of the reviewers' comments. If you do not agree with any of their criticisms or suggestions please explain clearly why this is so.

<u>Reviewer 1</u>

Advance summary and potential significance to field

In their manuscript "Iterative Bleaching Extends Multiplexity (IBEX) imaging facilitates simultaneous identification of all cell types in the vertebrate retina" Kothurkar and colleagues demonstrate the use of IBEX to visualize up to 11 antibodies on the same section of zebrafish retina, and show applicability of the technique in 2 other model organisms. They validate combination of IBEX with fluorescent reporter lines, fluorescent in situ hybridization and whole mount immunohistochemistry. They further use IBEX to reveal new insights into the coordinated development of retinal photoreceptors and Muller glial cells.

© 2024. Published by The Company of Biologists under the terms of the Creative Commons Attribution License (https://creativecommons.org/licenses/by/4.0/).

In the IBEX approach, primary antibodies are directly linked to fluorophores in order to overcome the difficulties of using multiple antibodies that were raised in the same species. To develop and validate their method, the authors demonstrate successful bleaching of fluorescent signals to enable multiple rounds of staining on the same section, and show simultaneous labeling with 4 distinctly labeled primary antibodies from the same species.

As the zebrafish is an increasingly important model organism for studying not only development, but also human diseases, expanding possibilities for multiplex labeling of tissue sections and whole mount samples, combining antibody with mRNA and transgene detection, represents a significant technical advancement. Overall this work presents a powerful technique, establishing the utility of IBEX for investigating cellular organization in highly complex tissues in the zebrafish as well as in other established and emerging (non-mammalian) vertebrate model organisms.

Comments for the author

While the paper is clearly written with well-presented data, there are a few points that should be clarified.

Line 141
For researchers studying zebrafish retina, it would be useful to indicate which specific antibodies required antigen retrieval. For these antibodies, does antigen retrieval also enhance signal for traditional staining (with secondary antibody)? Or is it only required for detecting directly conjugated antibodies? The temperature for antigen retrieval should be included in the methods (lines 456-457).

Figure 5 A''' — ” the vsx1 label is barely discernible
D''' —” Why is there significant non-overlapping signal between the antibody (PKC-beta) and in situ HCR detecting vsx1. These should be labeling the same (bipolar) cells?

Line 241 "there appear to be phagosomes in the RPE"
Can they be indicated in the figure (Figure S5) or otherwise shown (in a separate panel)?

Registration of the multiple images acquired seems an essential component of this approach, but the method is not clearly described. The authors refer to the Radtke 2020 publication, which mentions a 'SimpleITK workflow', but this is not described in detail. Radtke (2020) indicates that an Imaris XTension is used (thus expensive commercial software is required). The authors should include further details, if they use the Imaris XTension, and if there are any important parameters to consider.

Is there an open-source software that can perform the registration? If SimpleITK is used directly, this should be clarified and details provided.

For the whole mount samples, is registration performed on a projection or z-stack? This must be more challenging as the precise position for imaging is more difficult to replicate across rounds of imaging as compared to tissue sections. How is this managed?

IBEX is demonstrated for xenopus at tadpole stage and adult turquoise killifish. What about adult zebrafish retina? Have the authors tried stages beyond 5dpf? The authors should at least comment on whether it is expected to work, or what the technical limitations might be.

<u>Reviewer 2</u>

Advance summary and potential significance to field

In this technical manuscript, Korthukar et al. describe some adaptations of the IBEX method for multiplex immunofluorescence staining, initially reported by Radtke et al. in 2020, to be used on embryonic tissues, particularly testing its effectiveness on retinal tissue. Some of the relevant modifications introduced include the eventual quenching of some fluorophores resistant to LiHB4 with the antigen retrieval treatment (hot sodium citrate), the combination of rounds of

© 2024. Published by The Company of Biologists under the terms of the Creative Commons Attribution License (https://creativecommons.org/licenses/by/4.0/).

immunofluorescence with fluorescent protein reporter expression or fluorescent in situ hybridization, and its use on both tissue cryosections and whole-mount embryos. These improvements greatly increased the scope of the IBEX method to several new possible applications. They chose to apply the method to the retina, of zebrafish larvae and embryos, as well as of two other vertebrates, which is usually a rather problematic tissue because of antibody penetration issues (in whole mounts) and because of the difficulty to find appropriate cellular markers in these species. The list of antibodies successfully used, reported in the materials and methods section, is itself very useful for researchers in the field.

Comments for the author

The article is in general very well written, and the methods are well described. I highlight here only two minor issues that if revised by the authors will, in my opinion, improve the manuscript. The rest are just minor writing errors.

1- All along the manuscript there appears to be some confusion between the terms "bleaching" and "quenching". Since it is very clear that, when referred to fluorophores, they mean different things, it would be adequate to clarify why using one or the other, and, particularly, not to use them indistinctively. Since "quenching" would refer to a chemical reaction, usually causing a covalent modification of the fluorophore, inactivating it (in a reversible way at the appropriate conditions), both sodium citrate and LiBH4 would act as traditional "quenchers". I understand that LiBH4 quenching is performed under strong light conditions (another difference from the method described by Radtke et al.), which would add a more irreversible "photobleaching", and in this sense, the term "bleaching" could be appropriate. But this definitely needs clarification for the benefit of the readers.

2- In the "Materials and Methdos" section, "IBEX technique" subsection: the description of the LiBh4 treatment is missing the incubation time range.

3- Minor writing issues:
Ln. 67-68: The proper way of citing Santiago RamÃ³n y Cajal is by using the full surname, this is "RamÃ³n y Cajal".
Ln. 79: Check parentheses use in: "…(functional by 5 days post fertilisation (dpf)….
Ln. 137: IHC is "immunohistochemistry", usually reserved for the use of enzymes, such as peroxidase or phosphatase, to reveal antibody binding. In this case, it would be "immunofluorescence" or "IF".
Ln. 187: correct missing stop in " …Fig. 5B-B‴) We…".
Ln. 388: missing word in: "…it may be possible to >20 markers on a single zebrafish…".
Ln. 461: redaction in: "…were incubated with of the first round of antibodies…".
Ln. 656: correct "…tissue lost…", should be "loss". Immediately after, the fragment "…then tissue sectioned onto slides" is missing some connector to the rest of the sentence.
Several cases: separate the units from the value (ex.: "10 mM" instead of "10mM").

First revision

<u>Author response to reviewers' comments</u>

We greatly appreciate the reviewers comments and efforts to improve our manuscript. We have addressed each reviewer comment below.

Reviewer 1: **We thank reviewer one for the kind comments and suggestions for improvement. We have addressed each point below:**

The article is in general very well written, and the methods are well described. I highlight here only two minor issues that if revised by the authors will, in my opinion, improve the manuscript.

© 2024. Published by The Company of Biologists under the terms of the Creative Commons Attribution License (https://creativecommons.org/licenses/by/4.0/).

The rest are just minor writing errors.

1- All along the manuscript there appears to be some confusion between the terms "bleaching" and "quenching". Since it is very clear that, when referred to fluorophores, they mean different things, it would be adequate to clarify why using one or the other, and, particularly, not to use them indistinctively. Since "quenching" would refer to a chemical reaction, usually causing a covalent modification of the fluorophore, inactivating it (in a reversible way at the appropriate conditions), both sodium citrate and $LiBH_4$ would act as traditional "quenchers". I understand that $LiBH_4$ quenching is performed under strong light conditions (another difference from the method described by in the et al.), which would add a more irreversible "photobleaching", and in this sense, the term "bleaching" could be appropriate. But this definitely needs clarification for the benefit of the readers.

<u>Response:</u> The reviewer is correct. We have now defined when we are referring to photo-chemically inactivated or "bleached" or chemically "inactivated" or "quenched" (Lines 94-95 and 99-101). We have then been consistent throughout the remaining manuscript.

2- In the "Materials and Methods" section, "IBEX technique" subsection: the description of the LiBh4 treatment is missing the incubation time range.

<u>Response:</u> We have now added that this treatment is for 30 minutes (Line 523)

3- Minor writing issues:
Ln. 67-68: The proper way of citing Santiago Ramón y Cajal is by using the full surname, this is "Ramón y Cajal".

<u>Response:</u> We have corrected this in Line 65.

Ln. 79: Check parentheses use in: "…(functional by 5 days post fertilisation (dpf)…". Corrected

<u>Response:</u> We have deleted the additional parentheses in Line 76.

Ln. 137: IHC is "immunohistochemistry", usually reserved for the use of enzymes, such as peroxidase or phosphatase, to reveal antibody binding. In this case, it would be "immunofluorescence" or "IF".

<u>Response:</u> We have now corrected this to be immunofluorescence/IF throughout (Line 28).

Ln. 187: correct missing stop in " …Fig. 5B-B‴) We… ".

<u>Response:</u> We have corrected this (Line 191).

Ln. 388: missing word in: "…it may be possible to >20 markers on a single zebrafish…".

<u>Response:</u> It has now been corrected to "to use >20…". (Line 428).

Ln. 461: redaction in: "…were incubated with of the first round of antibodies…".

<u>Response:</u> We have removed "of" from this sentence (Line 513-514).

Ln. 656: correct "…tissue lost…", should be "loss". Immediately after, the fragment "…then tissue sectioned onto slides" is missing some connector to the rest of the sentence.

© 2024. Published by The Company of Biologists under the terms of the Creative Commons Attribution License (https://creativecommons.org/licenses/by/4.0/).

<u>Response:</u> **This has been corrected to "tissue loss, then tissue is sectioned onto slides" (Line 732).**

Several cases: separate the units from the value (ex.: "10 mM" instead of "10mM").

<u>Response:</u> **We have corrected this throughout.**

Reviewer 2: **We appreciate the reviewer highlighting that this is "clearly written and well-presented" and the work develops a "powerful technique, establishing the utility of IBEX for investigating cellular organization in highly complex tissues in the zebrafish as well as in other established and emerging (non-mammalian) vertebrate model organisms."**

Line 141
For researchers studying zebrafish retina, it would be useful to indicate which specific antibodies required antigen retrieval. For these antibodies, does antigen retrieval also enhance signal for traditional staining (with secondary antibody)? Or is it only required for detecting directly conjugated antibodies? The temperature for antigen retrieval should be included in the methods (lines 456-457).

<u>Response:</u> **We apologise for the confusion. In this study all tissues were treated with antigen retrieval before primary antibody incubation and directly conjugated antibodies (now stated in Methods Lines 509-511). We find that this enhances the staining by standard IF or IBEX. Additionally, initial heat treatment helps ensure that nuclear registration will be possible post the IBEX rounds, as heat treatment/antigen retrieval in later steps may cause the tissue to change shape. We have now included the temperature is approximately 100C (line 509).**

Figure 5 A''' — the vsx1 label is barely discernible

<u>Response:</u> **We have now increased the brightness of the image used in 5A'''.**

D''' — Why is there significant non-overlapping signal between the antibody (PKC- beta) and in situ HCR detecting vsx1. These should be labeling the same (bipolar) cells?

<u>Response:</u> **The reviewer is correct these do not perfectly overlap because vsx1 is a more general bipolar cell marker and PKC-Beta only labels the ON sub- class of bipolar cells. We have clarified in line 193, including additional references (Lines 189 and 193).**

Line 241 "there appear to be phagosomes in the RPE"
Can they be indicated in the figure (Figure S5) or otherwise shown (in a separate panel)?

<u>Response:</u> **We have now indicated them with an arrow in Supplemental Figure 5.**

Registration of the multiple images acquired seems an essential component of this approach, but the method is not clearly described. The authors refer to the Radtke 2020 publication, which mentions a 'SimpleITK workflow', but this is not described in detail. Radtke (2020) indicates that an Imaris XTension is used (thus expensive commercial software is required). The authors should include further details, if they use the Imaris XTension, and if there are any important parameters to consider.

<u>Response:</u> **We apologise for the confusion. The reference was incorrect and now been corrected to the more recent Radtke et al. 2022 (Line 591) which thoroughly details the registration process. We also clarified that this "free image viewer" can be used to rename files before using freely available 3D viewers or Fiji (Line 593).**

© 2024. Published by The Company of Biologists under the terms of the Creative Commons Attribution License (https://creativecommons.org/licenses/by/4.0/).

Is there an open-source software that can perform the registration? If SimpleITK is used directly, this should be clarified and details provided.

<u>Response:</u> **We have now clarified that you can indeed use a free viewer and provided these details (Lines 590-597).**

For the whole mount samples, is registration performed on a projection or z-stack? This must be more challenging as the precise position for imaging is more difficult to replicate across rounds of imaging as compared to tissue sections. How is this managed?

<u>Response:</u> **Yes, the reviewer is correct that it is more challenging. This was done by registering each z-section between rounds across the z-stack before building up the maximum projection. So precision is certainly key. However, the bleaching steps did not disturb tissue integrity to the point that we could not align multiple rounds in whole mount. Imaging parameters have now been expanded in the "IBEX for wholemount IF" section (Lines 565-567) and in the Image alignment section (Lines 586-587).**

IBEX is demonstrated for xenopus at tadpole stage and adult turquoise killifish. What about adult zebrafish retina? Have the authors tried stages beyond 5dpf? The authors should at least comment on whether it is expected to work, or what the technical limitations might be.

<u>Response:</u> **We have added a statement in the discussion saying that "as this technique has worked in multiple tissues, and we have tested in embryo and adult fish, we expect that this technique and the antibodies will work in adult zebrafish. Many of these antibodies have been used in adult tissues, as there is no difference in the treatment of the tissues we expect that they will work appropriately in adult zebrafish (Lines 356-359)**

Reviewer 3: **We appreciate the reviewer stating "I like this technique. I want to try IBEX! When I do, I will want to cite this paper.".**

The title claims that the authors have distinctly marked "all cell types" in the vertebrate retina. In some points within the text they are more careful and say things like "all major cell types" (e.g. Line 137) but in others the too-broad "all cell types" phrasing is used (e.g. Line 136). I think that a modifier is important throughout. The authors have been very comprehensive, but "all cell types" becomes falsifiable as soon as someone discovers a new retinal cell sub-type. Of course, "all cell types" are labeled (by DAPI, for instance), but the language sometimes implies that every type can be distinguished uniquely in the current paper.

<u>Response:</u> **We appreciate this comment and have modified the text throughout, including the title (Line 2), to highlight "all major cell types" (e.g. Line 31).**

Readers familiar with 'merely' 3-color RGB imaging are used to interpreting different hues as meaning "degrees of 3-color overlap" where Red+Blue=Magenta. Here, labels are overlaid with Magenta being its own channel, and the pattern comes from the fact that a Magenta cell is in a different position from the Red or Blue cell. People might understand the paper a little more intuitively if there were illustrations showing these cell types in different positions+colors next to both the merge and single- channel images. The existing figure 1A helps with this, but is hampered by the fact that there is no overlaid confocal image in this introductory figure (nor should there be). Could a similar merge illustration be added next to Figure 4A, with small single color schema added to the panels of 4B? The paper could benefit from similar illustrations on subsequent figures too, at least for the merge.

<u>Response:</u> **We appreciate this comment. We hope the reviewer can appreciate that working with so many cell labels and colours makes it challenging to represent in figures. We have now modified Figure 4A to show each round of antibody staining in cyan, magenta and yellow to visualise distinctions between labels. We have also included a representative schematic with colour for each round to demonstrate what cell type each label marks. Also, as these**

© 2024. Published by The Company of Biologists under the terms of the Creative Commons Attribution License (https://creativecommons.org/licenses/by/4.0/).

markers are used in follow on figure panels, we hope the reader will be able to follow. In Fig 5 and 6, in the merge we have now included which antibodies we use (5D, 6E). We hope the reviewer agrees this significant clarifies the staining data and presentation.

Did any unexpected cell sub-types come to light when the eye was labeled so comprehensively? Identification of even one unexpected labeling combination could be noteworthy.

Response: We agree with the reviewer. We are using "classic" markers for retinal cell types, and as the zebrafish retina is very well characterised, we did not observe any new sub-types. However, the characterisation of the killifish retina is not complete. We are following up on this with future studies, including the cell-type specific development and degeneration in the ageing killifish retina using IBEX. We have included a statement on this point in Lines 374-376 .

It would be nice to see some mention in discussion of whether the authors learned anything about how the zebrafish/Xenopus/killifish eyes compare using the IBEX technique. I recognize that this is not a comparative anatomy paper. Nonetheless, the paper would be stronger if the authors at least alluded to the power of this technique to do comparative anatomy.

Response: We again agree with the reviewer that this would be interesting but also not the point of the paper. We have included a statement to highlight that IBEX will be useful for comparative anatomy studies (Line 370-372).

There was a very recent paper showing that in situ HCR can be used to label 10 colors at once without bleaching, simply using spectral unmixing. That paper is so new that I don't think it diminishes the novelty of the current work. However, since the authors want readers to adopt the IBEX technique, they would be wise to comment on how the two methods differ and give examples of times when IBEX is preferential to spectral unmixing. I can think of a few, but it's best to spell things out for readers.

Response: We appreciate the reviewer pointing out this new paper. We have now included it as a reference and a statement highlighting the usefulness to explore cell morphology (by IF) and gene expression (by HCR) (Lines 331-333) and also how IBEX and spectral unmixing can be paired (Line 335-337).

Second decision letter

MS ID#: jcs.263407

MS TITLE: Iterative Bleaching Extends Multiplexity (IBEX) facilitates simultaneous identification of all major retinal cell types

AUTHORS: Aanandita A. Kothurkar, Gregory S. Patient, Nicole C. L. Noel, Aleksandra M. Krzywańska, Brittany J. Carr, Colin J. Chu and Ryan B. MacDonald

I am happy to tell you that your manuscript has been accepted for publication in Journal of Cell Science, pending standard publication integrity checks.

© 2024. Published by The Company of Biologists under the terms of the Creative Commons Attribution License (https://creativecommons.org/licenses/by/4.0/).
